# Decoding Polyether–Cation Interactions: Computational Strategies for Agricultural Applications

**DOI:** 10.3390/polym18070877

**Published:** 2026-04-02

**Authors:** João Vitor de Jesus Damante, Enzo Ernani da Silva, Felipe Breda Alves, Bruno Andrade Fico, Renato Luis Tame Parreira, Eduardo Ferreira Molina, Renato Pereira Orenha

**Affiliations:** Núcleo de Pesquisas em Ciências Exatas e Tecnológicas, Universidade de Franca, Franca 14404-600, SP, Brazil; damantejoaovitor@gmail.com (J.V.d.J.D.); enzoernanidasilva@gmail.com (E.E.d.S.); felipeb.alves@hotmail.com (F.B.A.); bruno.fico@outlook.com (B.A.F.); renato.parreira@unifran.edu.br (R.L.T.P.)

**Keywords:** polymer–cation interactions, agricultural micronutrients, DFT calculations, EDA–NOCV, polymeric nanogels

## Abstract

Zinc and iron are essential micronutrients in crop nutrition, and polymer-based nanogels have emerged as promising carriers to modulate their availability in sustainable agricultural systems. Here, a polymeric model receptor was designed to investigate how the nature and position of electron-donating (–NH_2_) and electron-withdrawing (–NO_2_) substituents control the recognition of Zn^2+^ and Fe^2+^ cations. Using a combination of density functional theory calculations, energy decomposition analysis with natural orbitals for chemical valence (EDA–NOCV), electrostatic potential (ESP) mapping, and quantum theory of atoms in molecules (QTAIM) method, the receptor–cation interactions are dissected into electrostatic, Pauli repulsion, orbital, and dispersion contributions. The results show that complex stability is governed mainly by orbital and electrostatic terms, with Fe^2+^ forming the most stable complex (−393.57 kcal mol^−1^) with regard to a Zn^2+^ similar complex (−288.80 kcal mol^−1^). Zn^2+^ complexes exhibit a broad tunability with substituent pattern. Electron-donating groups systematically strengthen both electrostatic and orbital components, whereas nitro substituents display a pronounced positional effect, ranging from strong destabilization to significant stabilization of Zn^2+^ binding. These findings establish molecular-level guidelines for engineering polymeric nanogels with tunable affinity and selectivity toward micronutrient cations in agricultural applications.

## 1. Introduction

Zinc is an essential micronutrient for plants, acting as a structural, catalytic, and regulatory cofactor in a wide range of enzymes and transcription factors. Adequate Zn(II) levels are crucial for processes such as auxin metabolism, protein synthesis, membrane integrity, and defense against oxidative stress. Zinc deficiency is one of the most widespread micronutrient disorders in crops, severely limiting yield and nutritional quality, particularly in cereals cultivated in calcareous or alkaline soils. To overcome this challenge, plants have evolved specific transport systems to take up and distribute Zn(II) within roots, shoots, and seeds, ensuring proper growth and reproduction [1,2,3].

Iron is another essential micronutrient for plant growth, being required for key processes such as photosynthesis, respiration, and chlorophyll biosynthesis [4,5]. Although iron is abundant in soils, it is often present in the form of insoluble Fe(III) oxides and hydroxides, which limits its bioavailability [6]. Most plants have therefore evolved strategies to acquire iron either by reducing Fe(III) to the more soluble Fe(II) at the root surface (Strategy I, used by dicotyledonous and non-graminaceous monocots), or by releasing phytosiderophores that chelate Fe(III) and facilitate its uptake (Strategy II, used by graminaceous species) [7]. Consequently, Fe(II) represents the main form absorbed and metabolized within plant tissues, while Fe(III) acts primarily as a reservoir in soils that must be chemically transformed before uptake [8].

Previous studies have investigated the interaction between polymers (or biopolymers) and cations through ionic complexation and cross-linking networks [9]. For instance, cation-induced gelation in alginate has been extensively studied, where metal ions such as Ca^2+^, Ba^2+^, Cu^2+^, Sr^2+^, Fe^2+^/Fe^3+^, and Al^3+^ promote the formation of “egg–box” structures that stabilize the polymer matrix [10]. The concentration of these metal ions strongly influences the gel properties, including density, thermal stability, diffusion resistance, and membrane-forming ability [11]. Furthermore, the affinity of alginate for different cations varies: Ca^2+^, Ba^2+^, Cd^2+^, Cu^2+^, Fe^3+^, and Al^3+^ exhibit higher binding affinities, while Mn^2+^, Co^2+^, Zn^2+^, and Ni^2+^ show weaker interactions [12]. These characteristics enable applications in tissue engineering, controlled drug delivery, and other biotechnology-related fields [9].

Atomistic molecular dynamics simulations of short poly(acrylate) chains in water revealed specific “site–binding” of Ca^2+^ to carboxylate groups and quantified how chain length/pH modulate coordination, offering a molecular picture of ion–polymer complexation that complements experimental trends [13]. In addition, density functional theory (DFT) modeling of aluminosilicate oligomers interacting with various cations—including Zn^2+^ and Fe^2+^—demonstrated that binding energies strongly correlate with ionic properties such as potential and radius, offering a promising pathway for the rational design of materials selective for specific ions [14].

**Figure 1:**
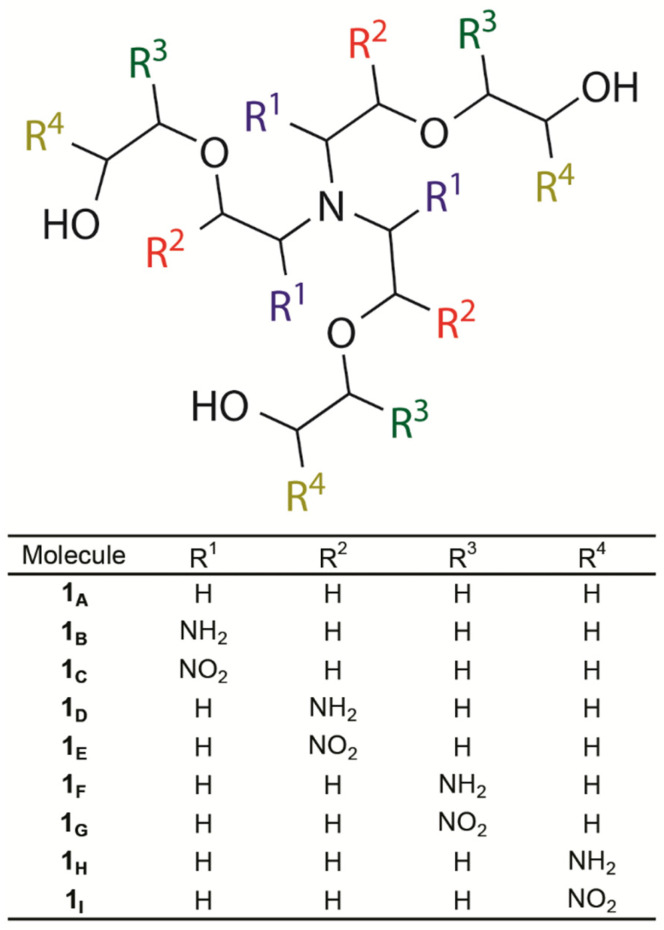
Structures of the substituted conformers: **1_A−I_**.

The development of polymers capable of interacting selectively with nutrient cations derived from metals, such as zinc and iron, has attracted growing interest, particularly in the context of sustainable agriculture, where these materials can act as controlled vectors or modulators of micronutrient availability in plants [15,16]. Despite their relevance, the mechanistic bases of these interactions remain poorly understood, especially regarding the structural modulation of the polymer to enhance its affinity for these ions. In this context, theoretical approaches emerge as essential tools for the rational design of such materials [17]. In this sense, the present polyether-based model, structurally derived from the nanogel system investigated in Ref. [17], is conceived as a simplified and chemically representative platform to isolate and rationalize fundamental ion–polymer interactions at the electronic level, while exploring interactions with different cations, emphasizing Zn(II) and Fe(II), essential nutrients for plant growth (Figure 1). In particular, we investigate how structural modifications replacing −H atoms with (i) electron-donating –NH_2_; or (ii) electron-withdrawing –NO_2_ groups in the molecular model modulate these interactions, aiming to enhance affinity and selectivity for Zn(II) or Fe(II) (Figure 1). The bonding interactions are examined through energy decomposition analysis (EDA) combined with the natural orbitals for chemical valence (NOCV) approach. The dominant electrostatic contributions are assessed by means of electrostatic potential (ESP) surface mapping, whereas the electron density distribution is further characterized via a topological study based on the quantum theory of atoms in molecules (QTAIM).

From a molecular perspective, ion recognition is increasingly understood as a multifactorial phenomenon in which binding strength and selectivity arise from the interplay between electrostatic attraction, polarization, and electronic structure effects. Computational investigations have shown that even subtle changes in the electronic environment of the binding site can lead to pronounced differences in ion affinity, highlighting the importance of electronic tuning as a general design principle for selective ion-binding systems [18,19,20].

## 2. Computational Methods

The molecular geometries were optimized without any structural constraints, and vibrational frequency calculations were carried out at the BP86 level [21,22] incorporating Grimme’s dispersion corrections with Becke–Johnson damping [D3(BJ)] [23] and the Def2–TZVP basis set [24]. To accelerate computations, the RIJCOSX approximation was applied [25,26], while Coulomb integrals were evaluated using the RI–J scheme [26] with the Def2/J auxiliary basis set [27]. Vibrational analyses were performed to confirm that all optimized structures correspond to true minima on the potential energy surface (absence of imaginary frequencies) at the chosen theoretical level. These calculations were conducted using the ORCA 6.0.0 package [28].

ESP surfaces and wavefunctions for QTAIM analysis were generated at the BP86–D3(BJ)/Def2–TZVP level employing Gaussian 16 (Revision A.03) [29]. The topological analysis of electron density was carried out with the QTAIM formalism [30] using AIMAll (Version 17.01.25) [31].

Chemical bonding interactions were further explored through the EDA [32]–NOCV [33,34] scheme, as implemented in the Amsterdam Density Functional (ADF) 2021 software [35,36] adopting the ZORA–BP86–D3(BJ) method together with the TZ2P basis set [37]. This theoretical framework (ZORA–BP86–D3(BJ)/TZ2P) proved suitable for elucidating the bonding mechanisms underlying noncovalent interactions [38,39].

Such an approach has been widely employed to provide qualitative and quantitative insight into the electronic factors underlying ion–ligand interactions, particularly when subtle energetic differences are responsible for observed trends in stability and selectivity [40].

For consistency, Fe^2+^ was treated in the low-spin configuration to allow a more direct comparison with the closed-shell Zn^2+^ complex, ensuring a balanced analysis of the electronic structure and bonding interactions within the same spin framework.

## 3. Results and Discussion

### 3.1. Cation Nature

Energy Decomposition Analysis was applied to clarify the bonding mechanism between, for instance, conformer **1_A_** and Zn^2+^. The interaction energy, Δ*E*_int_, is divided into four major contributions [32].

Δ*E*_int_ = Δ*V*_elstat_ + Δ*E*_Pauli_ + Δ*E*_oi_ + Δ*E*_disp_ (1)

The electrostatic contribution (Δ*V*_elstat_) describes the classical interaction between the charge distributions of the fragments before bond formation, reflecting the ionic or polar character of the interaction. The Pauli repulsion (Δ*E*_Pauli_) corresponds to the destabilizing term associated with the Pauli exclusion principle, which prevents the overlap of occupied orbitals and ensures the antisymmetry of the wavefunction. The orbital interaction (Δ*E*_oi_) includes both charge transfer and electronic polarization between the fragments, representing the covalent or partially covalent component of the bond. Finally, the Δ*E*_disp_ energy represents the dispersion corrections, following the approach proposed by Grimme and co-workers [23,41]. In this energy decomposition scheme, Δ*V*_elstat_ may be either attractive (negative energy values) or repulsive (positive energy values), depending on the charge distribution between the fragments. The Δ*E*_oi_ and Δ*E*_disp_ terms are intrinsically stabilizing and therefore always negative, whereas Δ*E*_Pauli_ is always positive, representing the destabilizing repulsion between occupied orbitals.

The EDA data for the interactions between the **1**_A–I_ conformers and cations (Zn^2+^ or Fe^2+^) are organized in Table 1. This method revealed that all complexes formed between the polymeric fragment and the Fe^2+^ or Zn^2+^ ions are stabilized by partially covalent bonds due to Δ*E*_oi_ (47–62%), Δ*V*_elstat_ (36–50%) and Δ*E*_disp_ (1–3%) contribution ranges in Δ*E*_oi_ + Δ*V*_elstat_ + Δ*E*_disp_ = 100%. Exceptionally, in the **1_E_**^….^Zn^2+^ interaction, there is a more than 100% contribution of Δ*E*_oi_ (107%) concerning to Δ*E*_oi_ + Δ*V*_elstat_ + Δ*E*_disp_ because of the repulsive Δ*V*_elstat_ energy (22.70 kcal mol^−1^).

The calculated interaction energies indicate that ion binding is not governed by a single dominant contribution, but rather by a combination of stabilizing factors whose relative importance depends on the chemical nature of the binding site. This observation is consistent with previous computational studies showing that ion–ligand interactions often deviate from a purely electrostatic description [42].

The Fe^2+^ ion establishes a more attractive interaction with the **1_A_** receptor concerning to Zn^2+^ cation, as can be visualized from the values of the Δ*E*_int_ energy in the **1_A_**^….^Fe^2+^ and **1_A_**^….^Zn^2+^ complexes (Table 1). It is supported from the more favorable Δ*V*_elstat_ and Δ*E*_oi_ energetic terms in the **1_A_**^….^Fe^2+^ interaction regarding the **1_A_**^….^Zn^2+^ bond. A more detailed analysis of the Δ*V*_elstat_ trends will be carried out through examination of the ESP surfaces. Moreover, the role of Δ*E*_oi_ will be assessed using the NOCV and QTAIM approaches.

**Figure 2:**
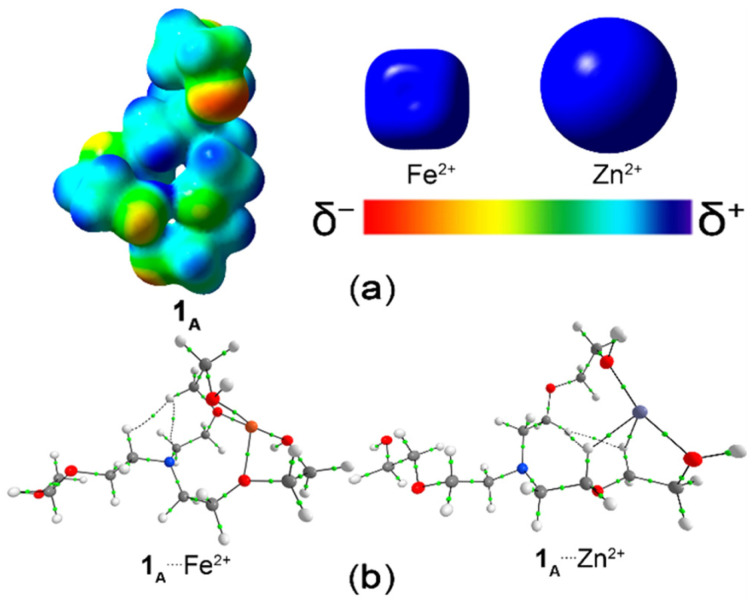
(**a**) Electrostatic potential surfaces mapped onto electronic densities of (i) 0.010 au [ranging from −0.010 au (red) to 0.010 au (blue)] for the **1_A_** receptor, and (ii) 0.020 au [ranging from 0.000 au (red) to 0.600 au (blue)] for Zn^2+^ and Fe^2+^ cations. (**b**) Topological map showing bond paths (continuous or dashed lines connecting the cores) and bond critical points (small light green points) for the **1_A_**^….^(Zn^2+^ or Fe^2+^) complexes. Color code for atoms: H = white, C = gray, N = blue, O = red, Fe = orange, and Zn = medium dark blue.

To rationalize the tendencies visualized in Δ*V*_elstat_, ESP maps of the isolated molecules **1_A_**_–**I**_ and the cations (Zn^2+^ and Fe^2+^) were obtained (Figure 2 and Figure S1). On these surfaces, red regions denote areas of high electron density (e.g., around nitrogen or oxygen atoms), whereas blue regions typically near hydrogen atoms indicate low electron density. The high electron density at the nitrogen or oxygen atoms of the isolated **1_A_**_–**I**_ structures suggest their propensity to interact with regions of low electron density in the isolated cations (Zn^2+^ or Fe^2+^). For a more quantitative assessment of the electrostatic interactions between **1_A_**_–**I**_ and the cations (Zn^2+^ or Fe^2+^) the minimum and maximum ESP values for the N/O atoms and the transition-metal cations, corresponding to the (N or O)^….^(Zn^2+^ or Fe^2+^) interactions, are compiled in Table 2.

The Fe^2+^ ion shows a more attractive electrostatic interaction with the **1_A_** compound regarding to Zn^2+^ cation. It occurs despite the lower ESP maximum value in Fe^2+^ concerning Zn^2+^. As shown in Figure 2, Fe^2+^ exhibits a more compact positive charge distribution in terms of its electronic density profile, which favors stronger electrostatic attraction with the oxygen donor atoms. This more localized charge distribution facilitates the formation of multiple O···cation interactions, as will be further confirmed by the QTAIM analysis.

The NOCV approach offers detailed insight into key orbital interactions, such as those occurring between **1_A_** and Zn^2+^, by decomposing the overall interaction into pairwise contributions from the most relevant molecular orbitals. Each pairwise orbital interaction associated with a given chemical bond can be visualized using deformation density channels, Δ*ρ*_k_(r), in which red regions indicate electron density depletion and blue regions indicate electron density accumulation. In addition, the NOCV method quantifies the energetic contribution (Δ*E*_oi,k_) of each density deformation channel (Δ*ρ*_k_) to the total orbital interaction energy (Δ*E*_oi_) [33,34].

**Figure 3:**
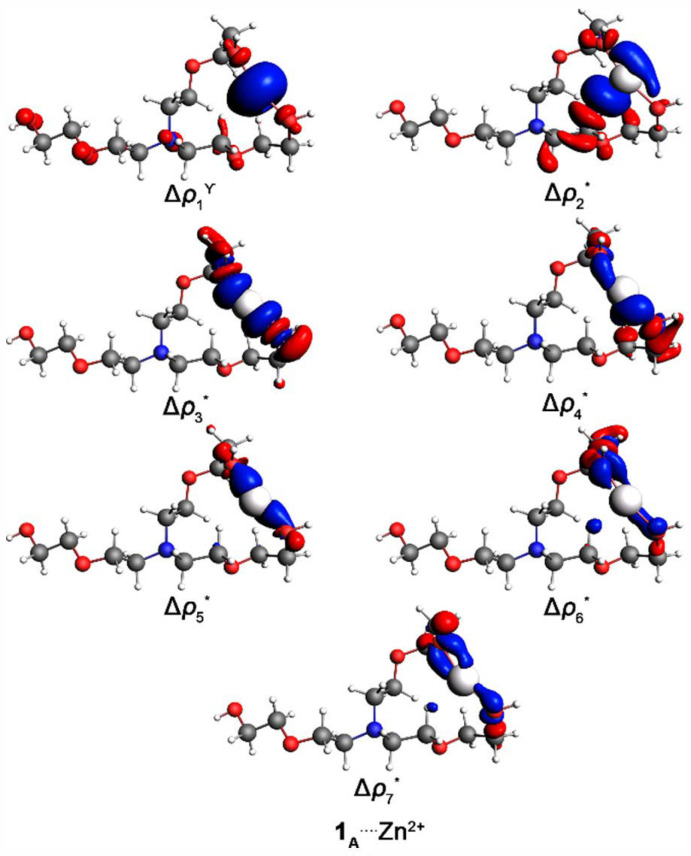
Surface plots of the first density deformation channels, Δ*ρ*_1–7_, with isovalues of * = 0.0010 and Υ = 0.0050 a.u. The red and blue regions represent electron density outflow and inflow, respectively, for the **1_A_**^….^Zn^2+^ complex.

The principal density deformation channels for the **1_A_**_–**I**_^….^(Zn^2+^ or Fe^2+^) complexes are shown in Figure 3 and Figures S2–S10, and the corresponding Δ*E*_oi,1–7_ values are summarized in Table 1. These channels reveal that the dominant orbital interactions in the **1_A_**_–**I**_^….^(Zn^2+^ or Fe^2+^) bonds are π interactions and, more predominantly, σ–type interactions involving (H, C, N or, chiefly, O)^….^(Zn^2+^ or Fe^2+^). Although the first two density deformation channels are repulsive in the **1_A_**^….^Fe^2+^ complex (Table 1), the remaining orbital interaction channels are significantly more stabilizing when compared to **1_A_**^….^Zn^2+^, indicating that the initial positive contributions arise from polarization-induced density rearrangements rather than genuine destabilizing interactions, and are effectively compensated by stronger donor–acceptor orbital interactions in subsequent channels. These stronger (N and especially O)^….^Fe^2+^ orbital interactions account for the overall more favorable Δ*E*_oi_ value observed for Fe^2+^ relative to Zn^2+^.

Additionally, a topological analysis of the electron density using the QTAIM approach identified bond critical points (BCPs) [43] between the **1_A_**_–**I**_ receptors and the cations (Zn^2+^ or Fe^2+^), as illustrated in Figure 2b and Figure S11 and summarized in Table S1. The ratio between the kinetic energy density (G_b_) and the potential energy density (V_b_), expressed as –G_b_/V_b_, at the BCPs associated with the (H, N or, predominantly O)^….^(Zn^2+^ or Fe^2+^) interactions falls within 0.5–1.0, supporting the partial covalent character of these bonds [44]. The sum of the electron density related to O^….^cation BCPs in the **1_A_**^….^Fe^2+^ complex is larger, in agreement with the more attractive Δ*E*_oi_ energy, than in the **1_A_**^….^Zn^2+^ structure (Table 1 and Table S1).

### 3.2. Modulation of the Ionic Recognition from Chemical Substitutions

Here, we propose electronic variations induced by substituents, such as, donor or withdrawing groups aiming modulate the strength of the receptor^….^cation bond (Figure 1). Donor substituents (−NH_2_) markedly enhance the overall stabilization, strengthening the electrostatic, orbital and dispersion components and yielding the most stable complexes in the series (**1_B, D, F or G_**^….^Zn^2+^ compared to **1_A_**^….^Zn^2+^). In addition, the presence of withdrawing groups (−NO_2_) also can contribute to a more favorable receptor^….^ion interaction. The −H → −NO_2_ substitutions in the −R^1^ or −R^3^ positions (**1_A_** → **1_C_** or **1_G_**, respectively) improve the Zn^2+^ recognition due to more attractive ΔV_elstat_, ΔE_oi_ and ΔE_disp_ energies, while that in the −R^4^ position (**1_A_** → 1_I_), it occurs from more favorable ΔE_oi_ energy and a less repulsive ΔE_Pauli_ component. On the other hand, the −H → −NO_2_ substitution in the −R^2^ position (**1_A_** → **1_E_**) disfavor the receptor–cation interaction because of a less attractive ΔE_disp_ energy term and chiefly from a repulsive ΔV_elstat_ energy.

Structural modifications can modulate the electrostatic interactions. Donor substituents (–NH_2_) tend to provide N coordinating sites containing minimum ESP values, and so intensify the electrostatic attraction in the **1**_(**B**,_ **_D_**_,_ **_F_** _or_ **_H_**)^….^Zn^2+^ complexes in relation to **1_A_**^….^Zn^2+^ molecule (Figure 2 and Figure S1, and Table 2). Electron-withdrawing substituents (–NO_2_) generate new O-coordinating sites containing minimum ESP values, and it promotes a more attractive electrostatic interactions in **1**_(**C** or_ **_G_**)^….^Zn^2+^ compared to **1_A_**^….^Zn^2+^ with the –H → –NO_2_ substitutions in –R^1^ and –R^3^ positions. On the other hand, the –H → –NO_2_ substitutions in –R^2^ and –R^4^ positions (generating **1_A_** → **1_E_** and **1_I_** molecules, respectively) are not followed by more favorable electrostatic **1**_(**E** or_ **_I_**_)_^….^Zn^2+^ in relation to **1_A_**^….^Zn^2+^. It can be explained from the larger ESP average values in the oxygen atoms associated with the ether group in O–cation interactions (Table 2). Additionally, in the **1_E_**^….^Zn^2+^ complex, the electrostatic repulsion (Δ*V*_elstat_ > 0) can also be partially attributed to the short spatial proximity between hydrogen atoms of the ligand and the Zn^2+^ cation, since both regions exhibit positive ESP values (Table 2). Importantly, this effect does not prevent complex stabilization, because electrostatic interactions represent only one component of the total interaction energy, which is strongly governed by attractive dispersion and, mainly, orbital contributions. It should be noted that the **1_E_**^….^Zn^2+^ structure may be further optimized toward a more stable geometry, potentially corresponding to a global minimum with a more typical coordination mode.

The NOCV analysis shows that the overall Δ*E*_oi_ term is not simply the sum of the individual orbital interaction contributions (Δ*E*_oi,1–7_) associated with the most significant density deformation channels (Δ*ρ*_1–7_) in the **1**_(**B**–**H**)_^….^Zn^2+^ substituted complexes relative to the **1_A_**^….^Zn^2+^ reference system. There is a more attractive Δ*E*_oi_ energy, but a less favorable Δ*E*_oi,1–7_ energy in the **1**_(**B**–**D** or_ **_F_**_–**H**)_^….^Zn^2+^ structures regarding the **1_A_**^….^Zn^2+^ molecule. It evidences that minority orbital interaction contributions are responsible for defining the more stable Δ*E*_oi_ energetic term in **1**_(**B**–**D** or_ **_F_**_–**H**)_^….^Zn^2+^ compared to **1_A_**^….^Zn^2+^. Exceptionally, in the –H → –NO_2_ substitutions in the –R^2^ and –R^4^ positions (**1_A_** → **1_E_** and **1_I_**, respectively), there are more attractive Δ*E*_oi_ along with Δ*E*_oi,1–7_ energies in the **1**_(**E** or_ **_I_**_)_^….^Zn^2+^ compounds than in the **1_A_**^….^Zn^2+^ complex. It appears in **1_E_**^….^Zn^2+^ concerning **1_A_**^….^Zn^2+^ due to π (C, N or chiefly O)^….^Zn^2+^ orbital interactions. These contributions, exhibiting π–type symmetry in the NOCV analysis, arise mainly from polarization and electrostatic effects associated with the Zn^2+^ cation, rather than genuine π–type covalent interactions, with possible minor cation–π contributions. Together, these effects constitute the main stabilizing component responsible for maintaining a favorable interaction in the **1_E_**^….^Zn^2+^ complex, even in the presence of localized electrostatic repulsion. In addition, it occurs in **1_I_**^….^Zn^2+^ regarding to **1_A_**^….^Zn^2+^ from π and, mainly, σ ONO^….^Zn^2+^ orbital interactions.

The QTAIM method shows that, in general, the sum of the electron density in the (H, N or principally O)^….^Zn^2+^ BCPs in the substituted complexes **1**_(**B**–**H**)_^….^Zn^2+^ is larger, so as the more attractive Δ*E*_oi_ energy, compared to reference structure: **1_A_**^….^Zn^2+^ (Table 1 and Table S1). As an exception, in the **1_E_**^….^Zn^2+^ molecule, there are only two H^….^Zn^2+^ BCPs, which shows a sum of the electron density relevantly lower regarding to (H or O)^….^Zn^2+^ BCPs associated to **1_A_**^….^Zn^2+^ compound. It can be explained by considering that the orbital interaction term (Δ*E*_oi_) in the **1_E_**^….^Zn^2+^ molecule is supported by enhanced long-range and π-type (C, N, and especially O)^….^Zn^2+^ contributions induced by the −NO_2_ substituent.

## 4. Conclusions

The integrated analysis of the EDA–NOCV, ESP, and QTAIM methods demonstrated that ionic recognition by the polymeric fragment is predominantly governed by orbital and electrostatic effects, which are modulated both by the nature of the cation and by the electronic substituents present on the ligand. The complex containing Fe^2+^ shows the most stable interaction in the series, supported by significantly more attractive orbital and electrostatic components, as well as a higher electron density in the bonding region compared with Zn^2+^. Among the Zn^2+^ derivatives, electron-donating substituents simultaneously enhance Δ*V*_elstat_ and Δ*E*_oi_, leading to more stable complexes. In contrast, electron-withdrawing substituents display strong positional dependence: while –NO_2_ groups at –R^1^, –R^3^, and –R^4^ reinforce stabilization, their insertion at –R^2^ induces pronounced electrostatic depolarization and increased repulsion, making the **1_E_**^….^Zn^2+^ complex the least favorable in the series. The NOCV channels confirm that structural changes adjust the balance between σ and π interactions, modulating receptor–cation orbital interaction. From a materials design perspective, these results indicate that the electronic nature and positional distribution of substituents along the polymer backbone can be strategically tuned to control cation affinity and selectivity. In practical terms, increasing electron-donating character near coordination sites may enhance nutrient retention, whereas careful positional placement of electron-withdrawing groups can prevent unfavorable electrostatic repulsion.

## Figures and Tables

**Table 1:** Analysis of the bonding situations between the polymeric fragments (**1_A–I_**) with the metal ions Zn^2+^ or Fe^2+^, obtained using the EDA–NOCV methodology ^a–c^.

Complex	∆*E*_int_	∆*V*_elstat_	∆*E*_Pauli_	∆*E*_oi_	∆*E*_disp_	∆*E*_oi,1_	∆*E*_oi,2_	∆*E*_oi,3_	∆*E*_oi,4_	∆*E*_oi,5_	∆*E*_oi,6_	∆*E*_oi,7_
**1_A_**^….^Fe^2+^	−393.57	−244.23 (37)	266.38	−406.33 (62)	−9.40 (1)	132.89	154.15	−234.94	−90.46	−80.06	−56.69	−28.72
**1_A_**^….^Zn^2+^	−288.80	−154.33 (39)	107.21	−232.56 (59)	−9.12 (2)	−94.33	−22.06	−27.30	−13.71	−12.25	−8.84	−7.57
**1_B_**^….^Zn^2+^	−412.76	−265.18 (49)	126.86	−259.70 (48)	−14.73 (3)	−67.39	−27.63	−23.49	−22.47	−10.35	−8.67	−7.03
**1_C_**^….^Zn^2+^	−365.61	−215.39 (45)	113.48	−252.71 (53)	−10.98 (2)	−59.84	−25.76	−21.48	−21.12	−12.89	−10.68	−9.60
**1_D_**^….^Zn^2+^	−356.33	−243.84 (50)	135.73	−238.42 (48)	−9.79 (2)	−72.40	−31.62	−24.91	−13.37	−11.26	−9.80	−8.21
**1_E_**^….^Zn^2+^	−248.51	22.70 (−9)	3.94	−271.17 (107)	−3.98 (2)	−256.11	−4.96	−1.29	−0.45	−1.77	−0.43	−0.24
**1_F_**^….^Zn^2+^	−412.81	−271.40 (50)	129.05	−256.67 (47)	−13.79 (3)	−68.84	−30.03	−25.83	−21.41	−10.98	−6.79	−7.20
**1_G_**^….^Zn^2+^	−335.63	−187.86 (41)	122.35	−256.14 (56)	−13.99 (3)	−68.84	−27.89	−22.26	−18.58	−11.56	−9.60	−7.25
**1_H_**^….^Zn^2+^	−410.77	−263.66 (50)	120.87	−254.79 (48)	−13.18 (2)	−71.35	−33.82	−24.33	−17.97	−11.55	−7.40	−6.42
**1_I_**^….^Zn^2+^	−324.23	−143.71 (36)	80.51	−251.68 (62)	−9.35 (2)	−102.35	−26.57	−26.67	−13.99	−12.12	−7.41	−6.79

^a^ The energy unit is kcal mol^−1^; ^b^ Δ*E*_int_ = Δ*V*_elstat_ + Δ*E*_Pauli_ + Δ*E*_oi_ + Δ*E*_disp_; ^c^ Values in parentheses represent the percentage of each stabilizing contribution (Δ*V*_elstat_ + Δ*E*_oi_ + Δ*E*_disp_ = 100%).

**Table 2:** Selected ESP values (kcal mol^−1^) for the isolated receptor structures (**1_A_**_–**I**_) and cations (Zn^2+^ and Fe^2+)^.

Structure	ESP_O_^a^	ESP_O_^OH b^	ESP_O_^NO2 c^	ESP_N_^NH2 d^	ESP_H_^e^	ESP_N_^central f^	ESP_Cation_^g^
**1_A_**	−21.08	−31.67	−	−	14.49	−	−
**1_B_**	−20.20	−31.49	−	−28.38	12.15	−	−
**1_C_**	0.77	−22.85	−31.44	−	21.21	−	−
**1_D_**	−24.50	−36.26	−	−29.36	8.90	−	−
**1_E_**	−18.18	−23.61	−23.17	−	−	−	−
**1_F_**	−14.25	−42.07	−	−30.40	3.36	−18.94	−
**1_G_**	−7.46	−24.95	−23.77	−	−	−7.46	−
**1_H_**	−20.20	−25.46	−	−25.40	−	−21.02	−
**1_I_**	−6.36	−27.92	−28.45	−	−	−	−
Zn^2+^	−	−	−	−	−	−	428.39
Fe^2+^	−	−	−	−	−	−	377.22

^a^ Average of the minimum ESP values present in the oxygen atoms associated with the ether group in O^….^cation. interaction; ^b^ Average of the minimum ESP values present in the oxygen atoms correlated to the HO^….^cation. interaction; ^c^ Average of the minimum ESP values present in oxygen atoms of the ONO^….^cation interaction; ^d^ Average of the minimum ESP values located in nitrogen of the −NH_2_ group related to the H_2_N^….^cation interaction; ^e^ Average minimum ESP values regarding to the hydrogen in H^….^cation interaction; ^f^ Average of the minimum ESP value belonging to nitrogen central atom, present in N^….^cation interaction; ^g^ Maximum ESP value located in the isolated cation.

## Data Availability

The original contributions presented in this study are included in the article/Supplementary Materials. Further inquiries can be directed to the corresponding authors.

## Supplementary Materials

The following supporting information can be downloaded at: https://www.mdpi.com/article/10.3390/polym18070877/s1, EDA–ESP–NOCV–QTAIM detailed analysis. ESP surfaces, density deformation channels (obtained from NOCV methodology), and QTAIM results to selected structures. Cartesian coordinates related to optimized geometry of all molecules investigated.

## Abbreviations

The following abbreviations are used in this manuscript:

EDA–NOCV	Energy Decomposition Analysis with Natural Orbitals for Chemical Valence
EDA	Energy Decomposition Analysis
NOCV	Natural Orbitals for Chemical Valence
ESP	Electrostatic Potential
QTAIM	Quantum Theory of Atoms in Molecules
DFT	Density Functional Theory
ADF	Amsterdam Density Functional
BCPs	Bond Critical Points
