# Supplementary material for: Decoding Polyether–Cation Interactions: Computational Strategies for Agricultural Applications
Source: Polymers (Basel). 2026 Apr 2;18(7):877. doi: 10.3390/polym18070877 (PMC13074813; doi:10.3390/polym18070877)
Supplement: Supplementary file 1 [file polymers-18-00877-s001.zip › polymers-4149566-supplementary.pdf]

Decoding Polyether–Cation Interactions: Computational Strategies for Agricultural Applications

João Vitor de Jesus Damante,[1] Enzo Ernani da Silva,[1] Felipe Breda Alves,[1] Bruno Andrade Fico,[1] Renato Luis Tame Parreira,[1] Eduardo Ferreira Molina,[1*] and Renato Pereira Orenha[1*]

[1] Núcleo de Pesquisas em Ciências Exatas e Tecnológicas, Universidade de Franca, 14404-600, Franca, SP, Brazil. Email: rorenha@unifran.edu.br / eduardo.molina@unifran.edu.br

Supplementary Material

Summary

	Page
**EDA–ESP–NOCV–QTAIM Analysis**	S3
**Figure S1.** Electrostatic potential surfaces mapped onto an electronic density of 0.010 a.u. [ranging from –0.100 a.u. (red) to 0.100 a.u. (blue)] for the $\mathbf{1}_{B–I}$ receptors.	S5
**Figure S2.** Surface plots of the first density deformation channels, $\Delta\rho_{1–7}$, with isovalues of * = 0.0010 and ϒ = 0.0050 a.u. The red and blue regions represent electron density outflow and inflow, respectively, for the $\mathbf{1}_{A}\cdots Fe^{2+}$ complex.	S6
**Figure S3.** Surface plots of the first density deformation channels, $\Delta\rho_{1–7}$, with isovalues of * = 0.0010 and ϒ = 0.0050 a.u. The red and blue regions represent electron density outflow and inflow, respectively, for the $\mathbf{1}_{B}\cdots Zn^{2+}$ complex.	S7
**Figure S4.** Surface plots of the first density deformation channels, $\Delta\rho_{1–7}$, with isovalues of χ = 0.0005, * = 0.0010 and ϒ = 0.0050 a.u. The red and blue regions represent electron density outflow and inflow, respectively, for the $\mathbf{1}_{C}\cdots Zn^{2+}$ complex.	S8
**Figure S5.** Surface plots of the first density deformation channels, $\Delta\rho_{1–7}$, with isovalues of * = 0.0010 and ϒ = 0.0050 a.u. The red and blue regions represent electron density outflow and inflow, respectively, for the $\mathbf{1}_{D}\cdots Zn^{2+}$ complex.	S9
**Figure S6.** Surface plots of the first density deformation channels, $\Delta\rho_{1–7}$, with isovalues of * = 0.0010 and ϒ = 0.0050 a.u. The red and blue regions represent electron density outflow and inflow, respectively, for the $\mathbf{1}_{E}\cdots Zn^{2+}$ complex.	S10
**Figure S7.** Surface plots of the first density deformation channels, $\Delta\rho_{1–7}$, with isovalues of χ = 0.0005, * = 0.0010 and ϒ = 0.0050 a.u. The red and blue regions represent electron density outflow and inflow, respectively, for the $\mathbf{1}_{F}\cdots Zn^{2+}$ complex.	S11
**Figure S8.** Surface plots of the first density deformation channels, $\Delta\rho_{1–7}$, with isovalues of χ = 0.0005, * = 0.0010 and ϒ = 0.0050 a.u. The red and blue regions represent electron density outflow and inflow, respectively, for the $\mathbf{1}_{G}\cdots Zn^{2+}$ complex.	S12

**Figure S9.** Surface plots of the first density deformation channels, $\Delta\rho_{1-7}$, with isovalues of χ = 0.0005, * = 0.0010 and Υ = 0.0050 a.u. The red and blue regions represent electron density outflow and inflow, respectively, for the $\mathbf{1}_{H}\cdots Zn^{2+}$ complex.	S13
**Figure S10.** Surface plots of the first density deformation channels, $\Delta\rho_{1-7}$, with isovalues of * = 0.0010 and Υ = 0.0050 a.u. The red and blue regions represent electron density outflow and inflow, respectively, for the $\mathbf{1}_{I}\cdots Zn^{2+}$ complex.	S14
**Figure S11.** Topological maps showing bond paths (continuous or dashed lines connecting the cores) and bond critical points (small light green points), for the $\mathbf{1}_{B-I}\cdots Zn^{2+}$ complexes. Color code for atoms: H = white, C = gray, N = blue, O = red and Zn = medium dark blue.	S15
**Table S1.** Ratio of kinetic energy density (G_b) to potential energy density (V_b), expressed as $-G_b/V_b$, along with the electron density (ϱ_b) at BCPs related to the interactions in the $\mathbf{1}_{A}\cdots Fe^{2+}$ and $\mathbf{1}_{A-I}\cdots Zn^{2+}$ complexes. All values are presented in atomic units (a.u.).	S16
**Table S2.** Optimized Cartesian coordinates for the compounds investigated in this study, calculated using the BP86–D3(BJ)/Def2–TZVP computational model.	S17

EDA–ESP–NOCV–QTAIM Analysis

To rationalize the trends in interaction energies, selectivity, and coordination behavior discussed in the *Results and Discussion* section of the main text, an integrated EDA–ESP–NOCV–QTAIM analysis was carried out. The purpose of this section is not to introduce new qualitative conclusions, but rather to provide a quantitative, electronic–level interpretation of the binding trends already established for Fe^{2+} and Zn^{2+} complexes and for the chemically substituted receptors.

Nature of the Cation: Comparison between Fe^{2+} and Zn^{2+}

As discussed in the main text, Fe^{2+} consistently exhibits a stronger interaction with the polymeric receptor than Zn^{2+}. This trend is quantitatively supported by the EDA results obtained for the reference complex **1**$_{\mathrm{A}}$. The total interaction energy is −393.57 kcal·mol^{-1} for **1**$_{\mathrm{A}}$···Fe^{2+}, whereas a substantially weaker interaction is found for **1**$_{\mathrm{A}}$···Zn^{2+} (−288.80 kcal·mol^{-1}), in full agreement with the stability ordering reported in Table 1 of the main article.

The EDA decomposition reveals that this difference originates from both electrostatic and orbital terms. The electrostatic contribution ($\Delta V_{\mathrm{elstat}}$) is markedly more attractive for Fe^{2+} (−244.23 kcal·mol^{-1}) than for Zn^{2+} (−154.33 kcal·mol^{-1}), reflecting a more efficient Coulombic interaction with the negatively charged regions of the receptor. In parallel, the orbital interaction term (ΔE_{oi}), associated with charge transfer and polarization, is also substantially larger for Fe^{2+} (−406.33 kcal·mol^{-1}) than for Zn^{2+} (−232.56 kcal·mol^{-1}). Dispersion contributes only marginally in both cases (≈1–2%), confirming that binding is dominated by electrostatic and orbital effects, as qualitatively described in the main text.

The ESP analysis provides a microscopic interpretation for these energetic trends. Although the isolated Zn^{2+} ion exhibits a higher maximum ESP value (428.39 kcal·mol^{-1}) than Fe^{2+} (377.22 kcal·mol^{-1}), the Fe^{2+} ion is able to approach the receptor more closely due to its smaller effective ionic radius. The receptor **1**$_{\mathrm{A}}$ presents strongly negative ESP regions localized on ether and hydroxyl oxygen atoms (minimum values down to −31.67 kcal·mol^{-1}), which enables Fe^{2+} to establish multiple short–range electrostatic contacts. This effect directly translates into the more stabilizing $\Delta V_{\mathrm{elstat}}$ observed in the EDA.

Consistent with this picture, the NOCV analysis shows that Fe^{2+} induces stronger orbital deformation channels than Zn^{2+}. In the **1**$_{\mathrm{A}}$···Fe^{2+} complex, several σ–type O → Fe^{2+} donation channels contribute very large stabilization energies, with individual $\Delta E_{\mathrm{oi,k}}$ values reaching up to −204 kcal·mol^{-1}. In contrast, the dominant O/N→Zn^{2+} channels in **1**$_{\mathrm{A}}$···Zn^{2+} are weaker, typically remaining below −190 kcal·mol^{-1}. These results provide an electronic–level explanation for the enhanced orbital contribution associated with Fe^{2+} binding discussed in the main text.

Finally, the QTAIM analysis corroborates the stronger and more cooperative nature of Fe^{2+} binding. Multiple bond critical points (BCPs) associated with O···Fe^{2+} interactions are identified, exhibiting relatively high electron densities (ρ_b up to 0.106 a.u.) and $-G_b/V_b$ ratios in the range 0.86–0.90, characteristic of interactions with partial covalent character. For Zn^{2+}, the corresponding BCPs show systematically lower electron densities (typically 0.03–0.10 a.u.), indicating weaker metal–ligand interactions. These topological descriptors fully support the energetic and orbital trends reported above and rationalize the higher affinity of Fe^{2+} highlighted in the main text.

Modulation of Ionic Recognition by Chemical Substitution

The modulation of Zn^{2+} binding by chemical substitution, described in the *Results and Discussion* section, was further analyzed at the electronic level for receptors $\mathbf{1}_{A\text{–}I}$ bearing electron–donating (–NH_2) and electron–withdrawing (–NO_2) groups. The EDA results quantitatively confirm that electron–donating substituents provide the strongest enhancement of metal binding. In particular, the –NH_2-substituted systems $\mathbf{1}_B$, $\mathbf{1}_F$, and $\mathbf{1}_H$ display total interaction energies of −412.76, −412.81, and −410.77 kcal·mol^{-1}, respectively, compared to −288.80 kcal·mol^{-1} for the unsubstituted complex $\mathbf{1}_A\cdots Zn^{2+}$, in agreement with the stability trend discussed in the main article.

This stabilization originates from a simultaneous reinforcement of electrostatic and orbital interactions. The electrostatic term becomes significantly more attractive in –NH_2–substituted systems, reaching ΔV_{elstat} values as low as −271.40 kcal·mol^{-1} ($\mathbf{1}_F\cdots Zn^{2+}$). The orbital interaction term is also enhanced, with ΔE_{oi} contributions ranging from −239 to −260 kcal·mol^{-1}, indicating increased charge donation and polarization toward the Zn^{2+} center.

The ESP analysis rationalizes these effects by revealing that –NH_2 groups introduce additional regions of strongly negative electrostatic potential, particularly on the nitrogen atoms (down to −30.40 kcal·mol^{-1}), which are spatially accessible to the metal ion. These regions reinforce long–range electrostatic attraction and facilitate the formation of additional coordination contacts. In contrast, –NO_2 substituents generate highly negative oxygen atoms, but their impact on Zn^{2+} binding is strongly position dependent. When favorably oriented toward the coordination cavity (as in $\mathbf{1}_C$ and $\mathbf{1}_G$), they contribute additional $O\cdots Zn^{2+}$ interactions, leading to enhanced stabilization (ΔE_{int} = −365.61 and −335.63 kcal·mol^{-1}, respectively). When unfavorably oriented, as in $\mathbf{1}_E$, the electrostatic contribution becomes repulsive (ΔV_{elstat} = +22.70 kcal·mol^{-1}), resulting in the weakest interaction of the series (ΔE_{int} = −248.51 kcal·mol^{-1}), as reported in the main text.

The NOCV analysis further shows that the increased orbital stabilization in substituted systems is not solely dictated by the dominant deformation channels ($\Delta\rho_1$–$\Delta\rho_7$). In several –NH_2–substituted receptors, the individual $\Delta E_{oi,k}$ values associated with the main channels are comparable to those of the unsubstituted system, indicating that the additional stabilization arises from a cumulative contribution of multiple weaker, more delocalized polarization and charge-transfer interactions. This finding provides a mechanistic explanation for the enhanced ΔE_{oi} values discussed in the main article.

Finally, the QTAIM analysis confirms that chemical substitution increases both the number and strength of metal–ligand interactions. –NH_2–substituted systems exhibit additional $N\cdots Zn^{2+}$ and $H_2N\cdots Zn^{2+}$ BCPs, with electron densities reaching up to 0.107 a.u. and $-G_b/V_b$ ratios between 0.80 and 0.85, indicative of partially covalent interactions. Nitro–substituted systems that stabilize Zn^{2+} show multiple $O\cdots Zn^{2+}$ BCPs, whereas the destabilized system $\mathbf{1}_E$ displays only weak $H\cdots Zn^{2+}$ contacts with low electron density. Overall, these topological features are fully consistent with the energetic trends and provide a robust electronic interpretation of the substitution effects discussed in the main text.

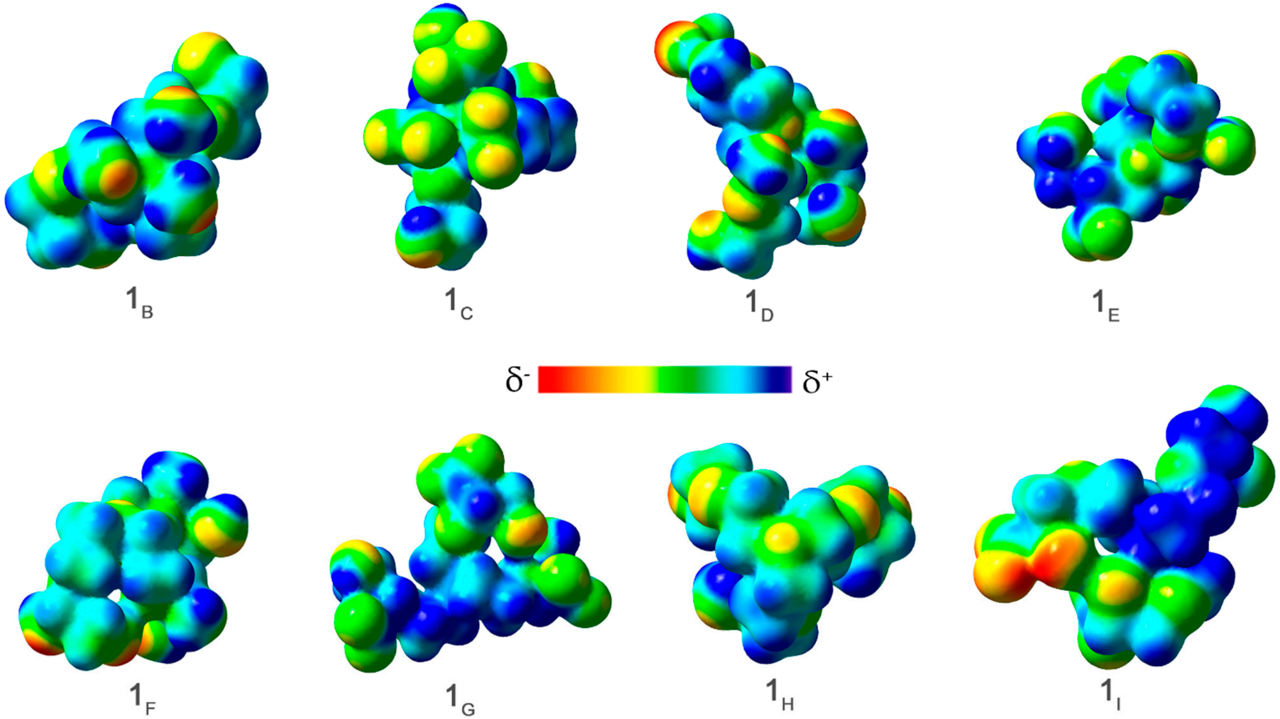

Figure S1. Electrostatic potential surfaces mapped onto an electronic density of 0.010 a.u. [ranging from −0.100 a.u. (red) to 0.100 a.u. (blue)] for the **1**$_{B\text{-}I}$ receptors.

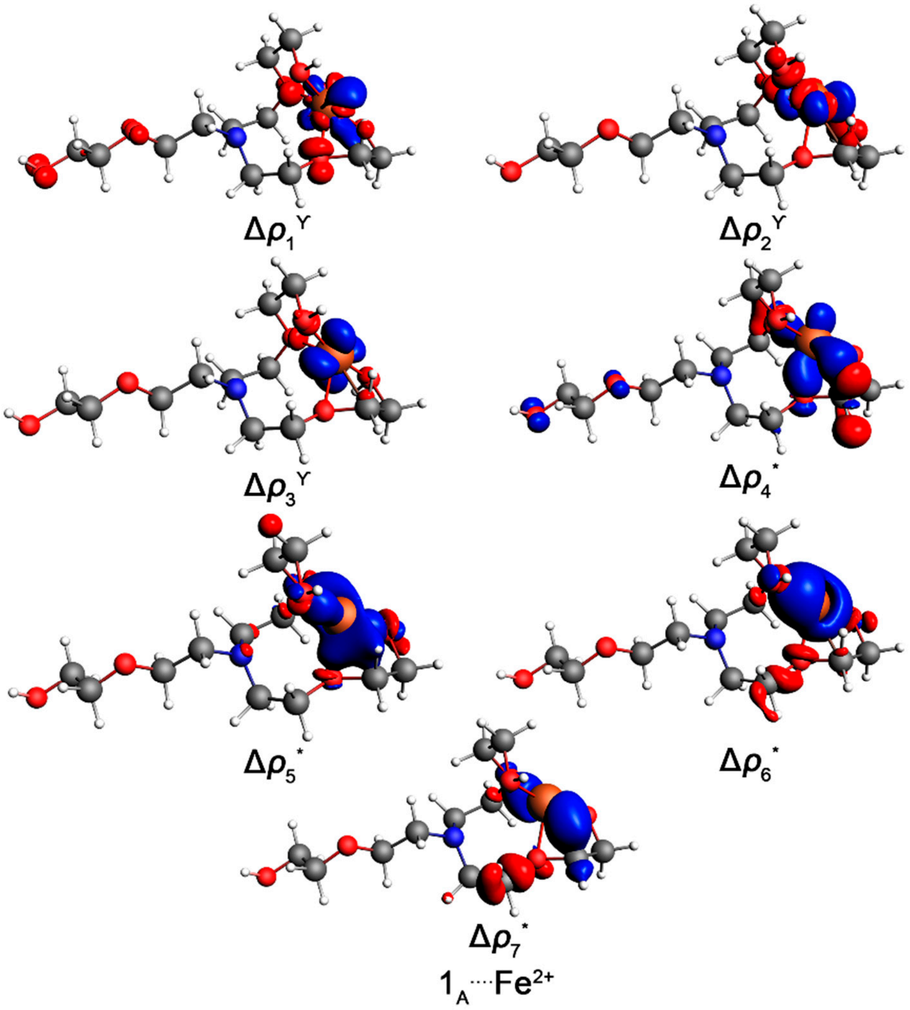

Figure S2. Surface plots of the first density deformation channels, $\Delta\rho_{1-7}$, with isovalues of * = 0.0010 and ϒ = 0.0050 a.u. The red and blue regions represent electron density outflow and inflow, respectively, for the $\mathbf{1}_{\mathrm{A}}\cdots Fe^{2+}$ complex.

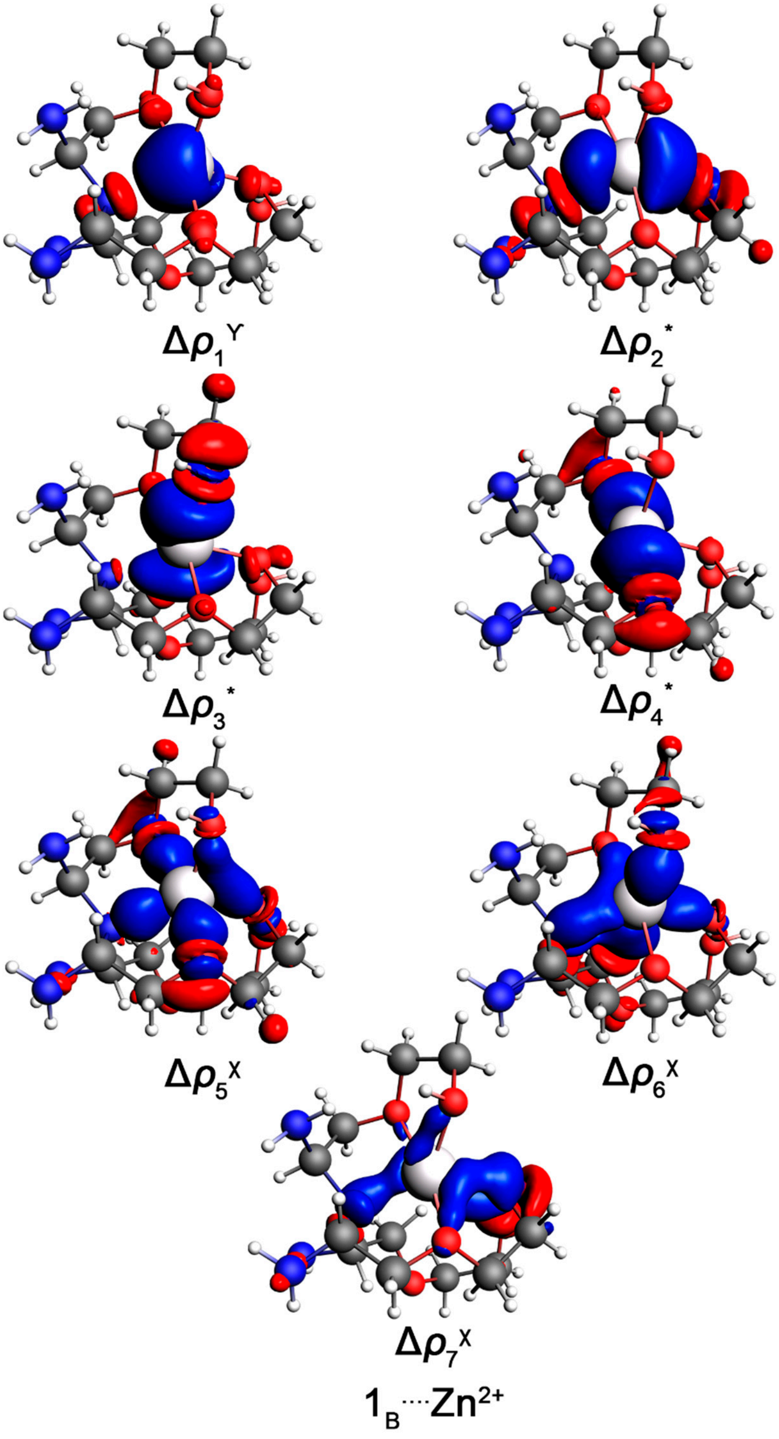

Figure S3. Surface plots of the first density deformation channels, $\Delta\rho_{1-7}$, with isovalues of χ = 0.0005, * = 0.0010 and ϒ = 0.0050 a.u. The red and blue regions represent electron density outflow and inflow, respectively, for the $\mathbf{1}_{B}\cdots Zn^{2+}$ complex.

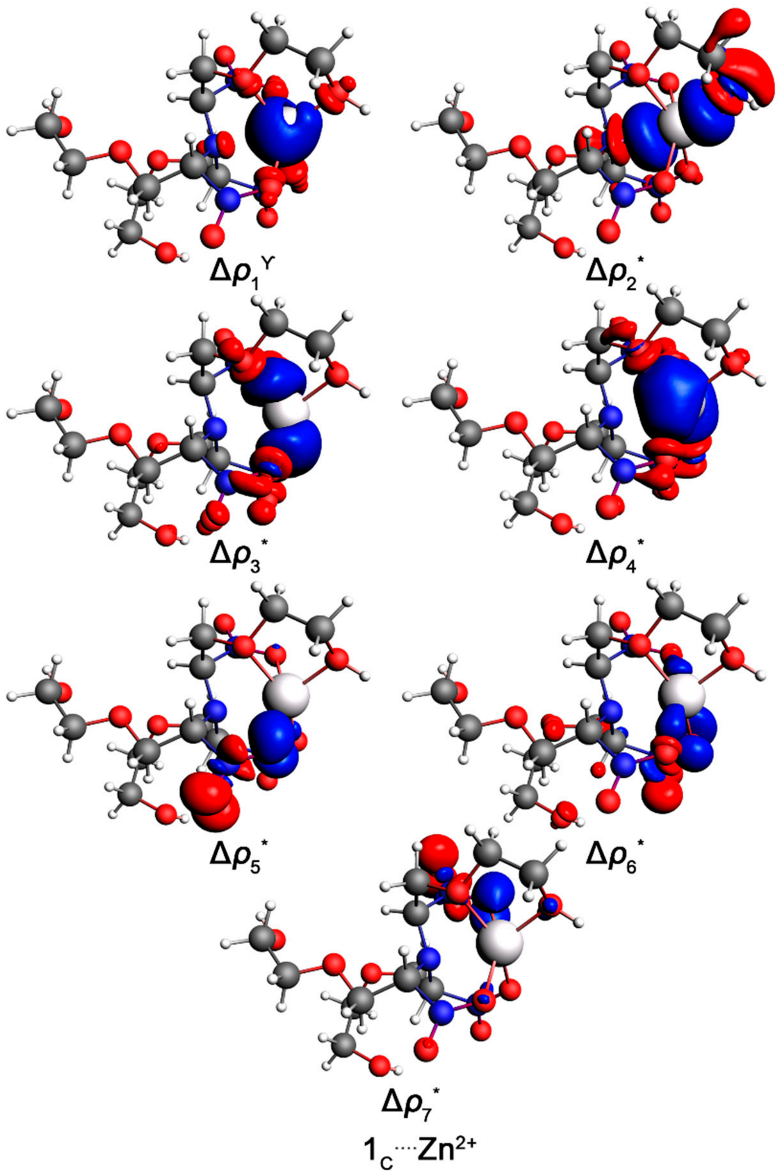

Figure S4. Surface plots of the first density deformation channels, $\Delta\rho_{1\text{–}7}$, with isovalues of * = 0.0010 and ϒ = 0.0050 a.u. The red and blue regions represent electron density outflow and inflow, respectively, for the **1**c····Zn^{2+} complex.

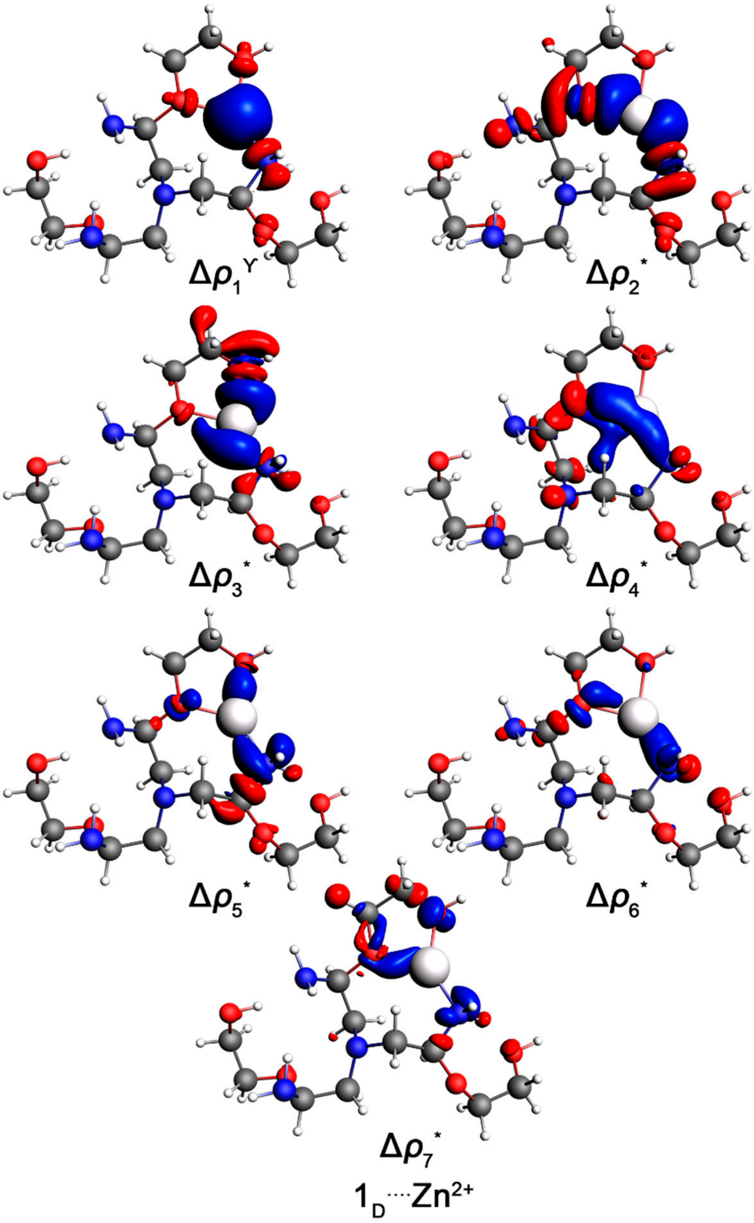

Figure S5. Surface plots of the first density deformation channels, $\Delta\rho_{1-7}$, with isovalues of * = 0.0010 and ϒ = 0.0050 a.u. The red and blue regions represent electron density outflow and inflow, respectively, for the $\mathbf{1_D}\cdots Zn^{2+}$ complex.

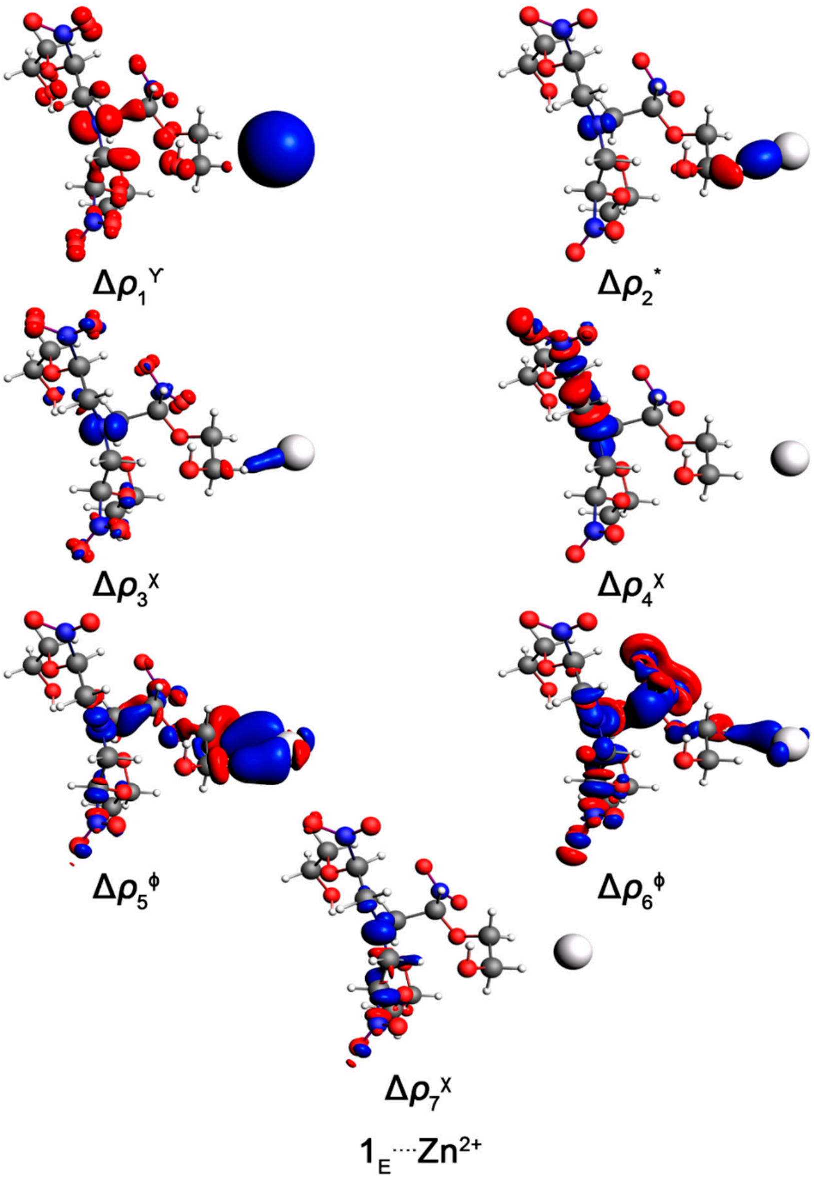

Figure S6. Surface plots of the first density deformation channels, $\Delta\rho_{1-7}$, with isovalues of ϕ = 0.0001, χ = 0.0005, * = 0.0010 and Υ = 0.0050 a.u. The red and blue regions represent electron density outflow and inflow, respectively, for the $\mathbf{1}_E\cdots Zn^{2+}$ complex.

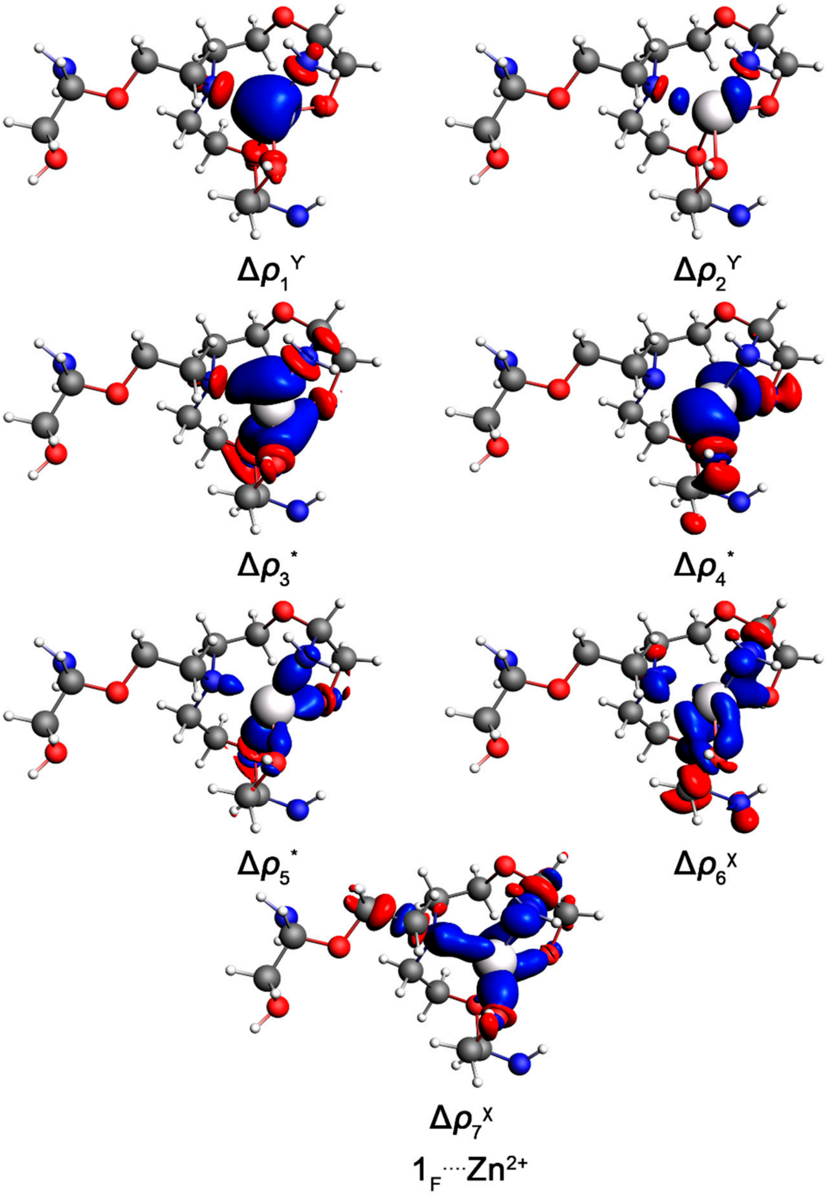

Figure S7. Surface plots of the first density deformation channels, $\Delta\rho_{1-7}$, with isovalues of χ = 0.0005, * = 0.0010 and ϒ = 0.0050 a.u. The red and blue regions represent electron density outflow and inflow, respectively, for the $\mathbf{1}_F\cdots Zn^{2+}$ complex.

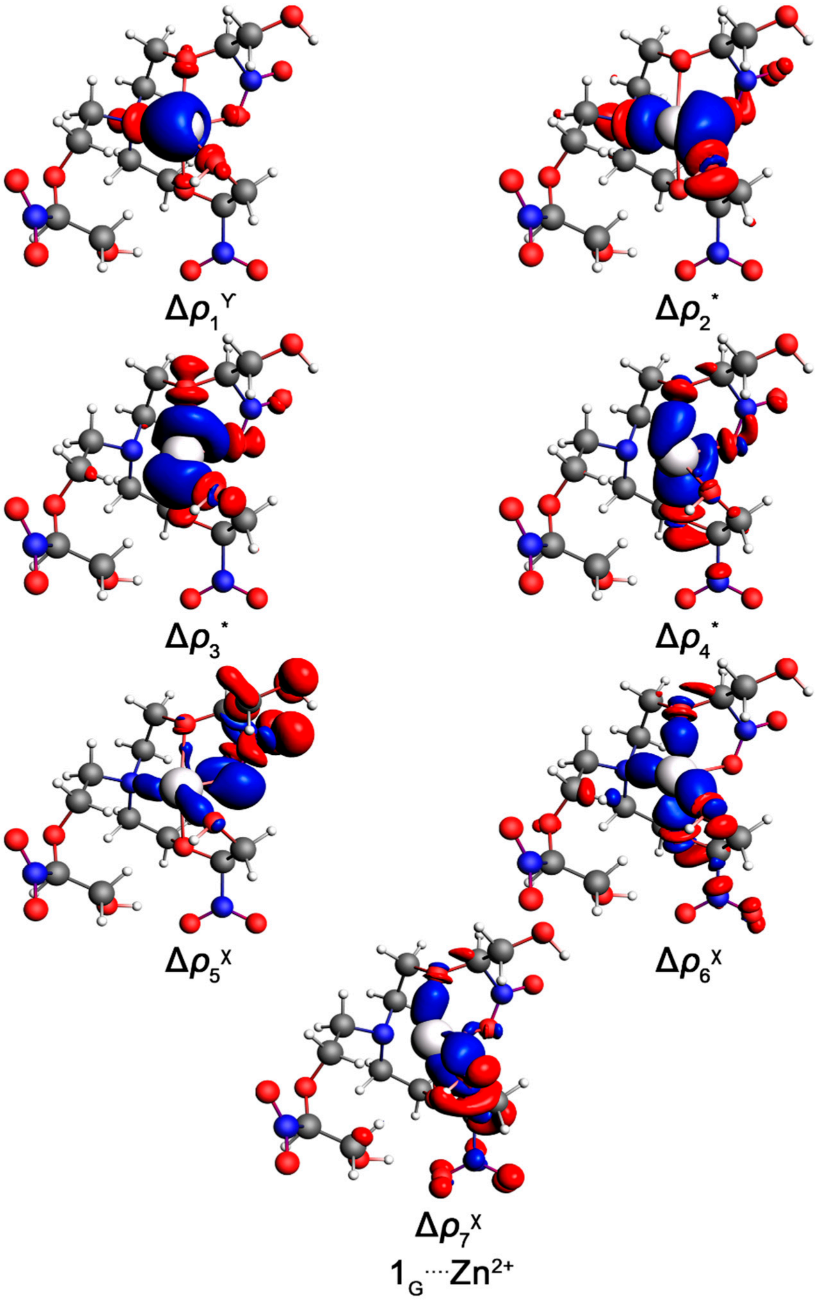

Figure S8. Surface plots of the first density deformation channels, $\Delta\rho_{1\text{–}7}$, with isovalues of χ = 0.0005, * = 0.0010 and ϒ = 0.0050 a.u. The red and blue regions represent electron density outflow and inflow, respectively, for the $\mathbf{1}_G\cdots Zn^{2+}$ complex.

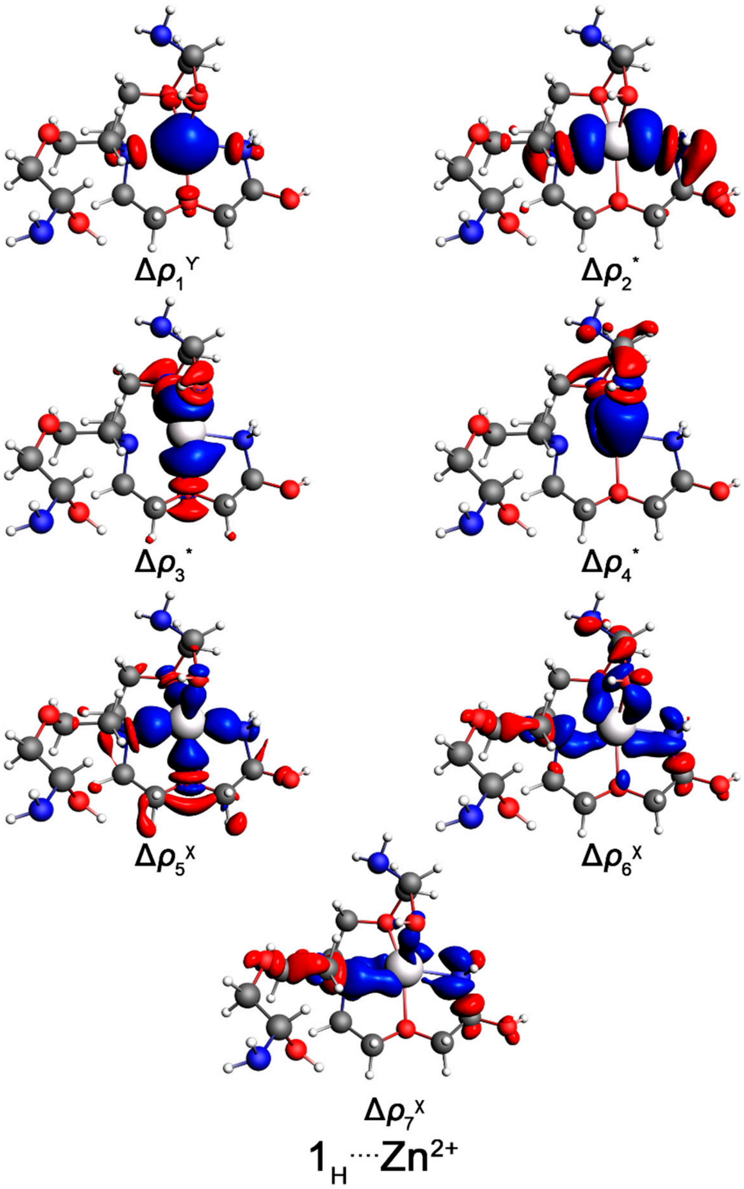

Figure S9. Surface plots of the first density deformation channels, $\Delta\rho_{1-7}$, with isovalues of χ = 0.0005, * = 0.0010 and ϒ = 0.0050 a.u. The red and blue regions represent electron density outflow and inflow, respectively, for the $\mathbf{1}_{H}\cdots Zn^{2+}$ complex.

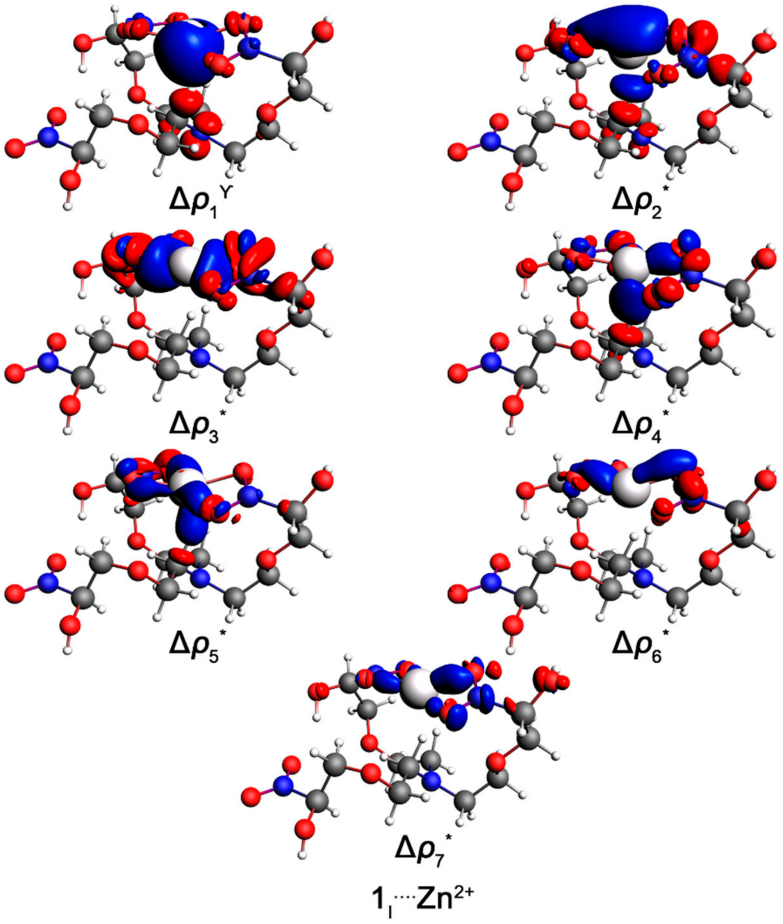

Figure S10. Surface plots of the first density deformation channels, $\Delta\rho_{1\text{–}7}$, with isovalues of * = 0.0010 and ϒ = 0.0050 a.u. The red and blue regions represent electron density outflow and inflow, respectively, for the $\mathbf{1}_{\mathbf{I}}\cdots Zn^{2+}$ complex.

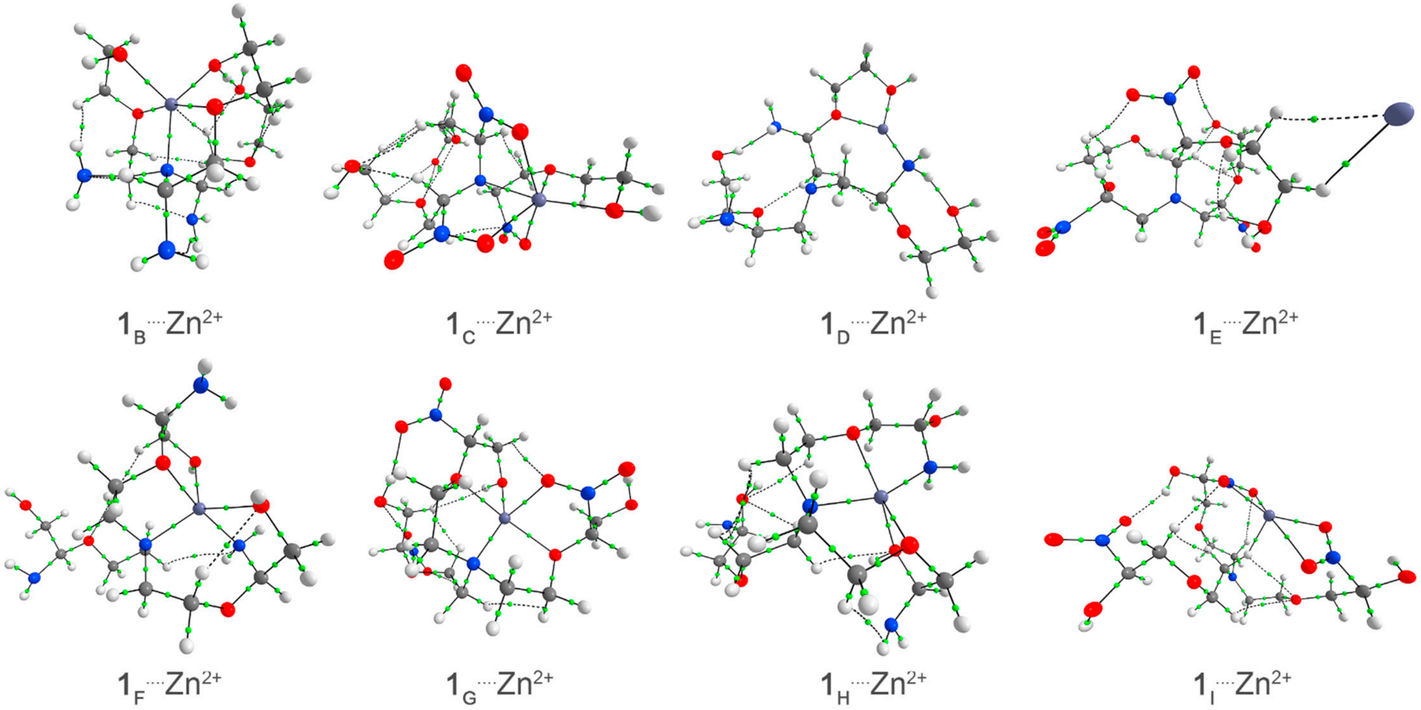

Figure S11. Topological maps showing bond paths (continuous or dashed lines connecting the cores) and bond critical points (small light green points), for the $\mathbf{1_{B\text{-}I}} \cdots Zn^{2+}$ complexes. Color code for atoms: H = white, C = gray, N = blue, O = red and Zn = medium dark blue.

Table S1. Ratio of kinetic energy density (G_b) to potential energy density (V_b), expressed as $–G_b/V_b$, along with the electron density (ϱ_b) at BCPs related to the interactions in the $\mathbf{1_A}{\cdots}Fe^{2+}$ and $\mathbf{1_{A–I}}{\cdots}Zn^{2+}$ complexes. All values are presented in atomic units (a.u.).

Complex	BCP	$–G_b/V_b$	ϱ_b
$\mathbf{1_A}{\cdots}Fe^{2+}$	$HO{\cdots}Fe^{2+}$	0.890	0.080
	$HO{\cdots}Fe^{2+}$	0.904	0.082
	$O{\cdots}Fe^{2+}$	0.896	0.095
	$O{\cdots}Fe^{2+}$	0.864	0.106
$\mathbf{1_A}{\cdots}Zn^{2+}$	$HO{\cdots}Zn^{2+}$	0.869	0.102
	$HO{\cdots}Zn^{2+}$	0.867	0.104
	$H{\cdots}Zn^{2+}$	0.760	0.046
	$H{\cdots}Zn^{2+}$	0.804	0.029
$\mathbf{1_B}{\cdots}Zn^{2+}$	$HO{\cdots}Zn^{2+}$	0.887	0.076
	$HO{\cdots}Zn^{2+}$	0.901	0.066
	$O{\cdots}Zn^{2+}$	0.898	0.061
	$O{\cdots}Zn^{2+}$	0.898	0.059
	$N{\cdots}Zn^{2+}$	0.833	0.078
	$H{\cdots}Zn^{2+}$	0.887	0.019
$\mathbf{1_C}{\cdots}Zn^{2+}$	$HO{\cdots}Zn^{2+}$	0.890	0.079
	$O{\cdots}Zn^{2+}$	0.913	0.054
	$ONO{\cdots}Zn^{2+}$	0.914	0.054
	$ONO{\cdots}Zn^{2+}$	0.902	0.066
	$ONO{\cdots}Zn^{2+}$	0.909	0.060
	$N{\cdots}Zn^{2+}$	0.866	0.050
$\mathbf{1_D}{\cdots}Zn^{2+}$	$HO{\cdots}Zn^{2+}$	0.881	0.084
	$O{\cdots}Zn^{2+}$	0.878	0.085
	$H_2N{\cdots}Zn^{2+}$	0.804	0.107
$\mathbf{1_E}{\cdots}Zn^{2+}$	$H{\cdots}Zn^{2+}$	0.790	0.028
	$H{\cdots}Zn^{2+}$	0.951	0.015
$\mathbf{1_F}{\cdots}Zn^{2+}$	$HO{\cdots}Zn^{2+}$	0.918	0.055
	$HO{\cdots}Zn^{2+}$	0.902	0.068
	$O{\cdots}Zn^{2+}$	0.896	0.060
	$N{\cdots}Zn^{2+}$	0.835	0.075
	$H_2N{\cdots}Zn^{2+}$	0.850	0.081
$\mathbf{1_G}{\cdots}Zn^{2+}$	$HO{\cdots}Zn^{2+}$	0.885	0.076
	$O{\cdots}Zn^{2+}$	0.909	0.054
	$O{\cdots}Zn^{2+}$	0.908	0.055
	$ONO{\cdots}Zn^{2+}$	0.911	0.055
	$N{\cdots}Zn^{2+}$	0.819	0.089
$\mathbf{1_H}{\cdots}Zn^{2+}$	$HO{\cdots}Zn^{2+}$	0.911	0.061
	$O{\cdots}Zn^{2+}$	0.899	0.063
	$O{\cdots}Zn^{2+}$	0.897	0.055
	$N{\cdots}Zn^{2+}$	0.833	0.074
	$H_2N{\cdots}Zn^{2+}$	0.848	0.079
$\mathbf{1_I}{\cdots}Zn^{2+}$	$ONO{\cdots}Zn^{2+}$	0.919	0.045
	$ONO{\cdots}Zn^{2+}$	0.882	0.069
	$ONO{\cdots}Zn^{2+}$	0.930	0.033
	$ONO{\cdots}Zn^{2+}$	0.879	0.075
	$H{\cdots}Zn^{2+}$	0.753	0.062

Table S2. Optimized Cartesian coordinates for the compounds investigated in this study, calculated using the BP86–D3(BJ)/Def2–TZVP computational model.

1A

N	-1.202904000	0.694932000	0.743180000
C	-0.085660000	-0.168581000	1.152880000
C	-0.117835000	-1.607650000	0.624868000
O	0.800588000	-2.422127000	1.378727000
C	2.013996000	-2.794385000	0.711109000
C	3.046398000	-1.686135000	0.520815000
O	2.955601000	-0.956843000	-0.719641000
C	-2.507271000	0.268200000	1.301961000
C	-3.399632000	1.447219000	1.687972000
O	-3.262797000	1.883684000	3.055641000
C	-1.938326000	2.214705000	3.510115000
C	-1.305629000	3.442501000	2.873507000
O	-0.759121000	3.237145000	1.563831000
C	-1.318920000	0.918216000	-0.712120000
C	-0.068636000	1.507547000	-1.359787000
O	0.877140000	0.535765000	-1.845648000
C	0.440119000	-0.093523000	-3.065532000
C	1.554056000	-0.928311000	-3.663270000
O	1.835268000	-2.136154000	-2.941662000
H	-0.095805000	-0.209353000	2.252830000
H	0.854354000	0.324812000	0.867032000
H	0.122922000	-1.654732000	-0.448205000
H	-1.120224000	-2.040183000	0.769091000
H	1.786015000	-3.262578000	-0.263342000
H	2.458709000	-3.561722000	1.362074000
H	4.042342000	-2.151546000	0.501970000
H	3.010661000	-0.992033000	1.376797000
H	2.155055000	-0.381560000	-0.747768000
H	-2.331007000	-0.351176000	2.192930000
H	-3.048682000	-0.364776000	0.576707000
H	-4.453464000	1.148211000	1.607818000

H	-3.236847000	2.293638000	0.998278000
H	-1.263686000	1.344468000	3.415721000
H	-2.063824000	2.415736000	4.584001000
H	-0.497470000	3.776232000	3.550822000
H	-2.054347000	4.254517000	2.835359000
H	-0.895586000	2.258366000	1.300441000
H	-2.143102000	1.631659000	-0.857504000
H	-1.616506000	-0.008925000	-1.237098000
H	0.481216000	2.130137000	-0.640661000
H	-0.370829000	2.148137000	-2.205240000
H	0.155924000	0.692901000	-3.789142000
H	-0.444264000	-0.727004000	-2.878270000
H	2.466225000	-0.313340000	-3.761529000
H	1.238994000	-1.226075000	-4.674335000
H	2.283808000	-1.860789000	-2.098713000

1B

N	0.884904000	1.346169000	-0.467499000
C	2.083510000	0.696432000	-0.951009000
C	2.433716000	-0.496726000	-0.049808000
O	3.529589000	-1.244579000	-0.614966000
C	3.253126000	-2.631218000	-0.862328000
C	2.467084000	-2.929142000	-2.133435000
O	1.036112000	-2.819265000	-2.033470000
C	0.951743000	2.031169000	0.828558000
C	-0.090535000	1.541517000	1.833997000
O	-1.397785000	2.022644000	1.484717000
C	-2.468177000	1.376097000	2.185084000
C	-2.744608000	1.920087000	3.576118000
O	-1.738644000	1.596041000	4.555300000
C	-0.125277000	1.754592000	-1.431447000
C	-0.584892000	0.579615000	-2.324765000
O	-0.622335000	-0.690467000	-1.678383000
C	-1.696684000	-0.847393000	-0.735810000
C	-1.360928000	-1.954629000	0.243289000

O	-1.196432000	-3.245161000	-0.367407000
N	0.203822000	2.837309000	-2.383521000
N	0.940976000	3.505378000	0.810849000
N	3.228516000	1.622787000	-1.152606000
H	1.852933000	0.279672000	-1.941322000
H	1.551183000	-1.140097000	0.064601000
H	2.741501000	-0.145901000	0.950716000
H	2.746696000	-3.082323000	0.009482000
H	4.241399000	-3.105317000	-0.966145000
H	2.661081000	-3.978128000	-2.403955000
H	2.840022000	-2.293781000	-2.954728000
H	0.743528000	-1.878053000	-1.970674000
H	1.909433000	1.727154000	1.274591000
H	0.189009000	1.899148000	2.838402000
H	-0.093941000	0.438451000	1.843804000
H	-2.278397000	0.287117000	2.235956000
H	-3.369317000	1.535527000	1.572893000
H	-3.731742000	1.538835000	3.897633000
H	-2.796913000	3.018009000	3.547631000
H	-1.634290000	0.626184000	4.547721000
H	-0.976822000	2.106527000	-0.837349000
H	0.116513000	0.457602000	-3.163538000
H	-1.573194000	0.824081000	-2.748470000
H	-2.627994000	-1.076920000	-1.284965000
H	-1.845508000	0.085015000	-0.168771000
H	-2.195191000	-2.041811000	0.954985000
H	-0.457089000	-1.682845000	0.815571000
H	-0.345568000	-3.224975000	-0.870031000
H	1.061437000	2.571895000	-2.878107000
H	0.438522000	3.679100000	-1.852243000
H	0.022394000	3.823943000	0.489100000
H	1.614942000	3.827326000	0.111097000
H	4.025006000	1.054513000	-1.463037000
H	3.508476000	1.981019000	-0.231688000

1c

N	-0.738051000	-1.012818000	-1.532584000
C	-1.671519000	-0.019895000	-1.103586000
C	-2.083777000	1.022651000	-2.148682000
O	-0.888971000	1.592181000	-2.649692000
C	-1.127566000	2.510517000	-3.733310000
C	0.132070000	2.603329000	-4.565859000
O	0.388153000	1.411978000	-5.322137000
C	-0.862961000	-1.608380000	-2.817725000
C	0.450340000	-1.847508000	-3.589269000
O	1.169872000	-0.621496000	-3.596533000
C	2.609318000	-0.740896000	-3.570341000
C	3.222770000	-1.017641000	-4.922258000
O	2.932308000	-2.370053000	-5.321007000
C	0.370408000	-1.342303000	-0.696724000
C	1.195040000	-0.183480000	-0.135881000
O	0.486800000	0.458286000	0.917970000
C	1.154763000	1.648827000	1.367994000
C	0.372177000	2.210052000	2.532645000
O	0.385293000	1.344762000	3.681709000
N	-0.054832000	-2.212516000	0.538611000
O	-1.231898000	-2.206038000	0.885025000
O	0.850402000	-2.817840000	1.111856000
N	-1.535072000	-3.023202000	-2.761687000
O	-1.287970000	-3.737560000	-1.792707000
O	-2.234266000	-3.345583000	-3.722246000
N	-3.018136000	-0.641405000	-0.614113000
O	-3.596147000	-0.060987000	0.304658000
O	-3.439044000	-1.640078000	-1.195111000
H	-1.272309000	0.475678000	-0.213338000
H	-2.719211000	1.772522000	-1.647046000
H	-2.675199000	0.569045000	-2.963890000
H	-1.400496000	3.502773000	-3.331341000
H	-1.961963000	2.140565000	-4.354301000

H	0.990625000	2.846622000	-3.914977000
H	0.013001000	3.423979000	-5.288297000
H	0.622428000	0.691007000	-4.684501000
H	-1.555866000	-1.027979000	-3.431257000
H	0.189284000	-2.171474000	-4.606965000
H	1.046767000	-2.645249000	-3.120467000
H	2.891973000	-1.517664000	-2.840086000
H	2.972487000	0.230664000	-3.209129000
H	4.313521000	-0.861008000	-4.837030000
H	2.828041000	-0.300254000	-5.661131000
H	3.232439000	-2.471614000	-6.242004000
H	1.052239000	-2.007266000	-1.231969000
H	1.389468000	0.512059000	-0.969664000
H	2.157647000	-0.576985000	0.229754000
H	1.194631000	2.388571000	0.548239000
H	2.187136000	1.399246000	1.671649000
H	-0.665419000	2.418190000	2.218244000
H	0.833280000	3.156738000	2.845795000
H	-0.030178000	0.506093000	3.407809000

1D

N	-0.618176000	-0.033308000	0.605517000
C	0.423638000	-0.083535000	1.630163000
C	1.061729000	-1.460524000	1.831581000
O	2.002698000	-1.685696000	0.736557000
C	2.996729000	-2.686052000	1.014623000
C	3.135717000	-3.603331000	-0.182204000
O	1.933968000	-4.355521000	-0.427932000
C	-1.706737000	0.905302000	0.875254000
C	-1.388295000	2.406050000	0.799035000
O	-0.569360000	2.738706000	1.917009000
C	0.258742000	3.911143000	1.762581000
C	-0.424437000	5.205181000	2.172401000
O	-1.447698000	5.633363000	1.266959000

C	-0.130130000	0.022601000	-0.777698000
C	-0.559695000	-1.168191000	-1.639124000
O	0.148253000	-1.098132000	-2.914982000
C	1.464286000	-1.666995000	-2.863342000
C	2.123486000	-1.531831000	-4.211806000
O	2.403400000	-0.139604000	-4.465707000
N	-1.976917000	-1.164657000	-1.863997000
N	-2.609112000	3.232437000	0.800715000
N	0.062680000	-2.490011000	1.969301000
H	-0.038822000	0.186381000	2.587957000
H	1.239742000	0.641157000	1.448481000
H	1.655817000	-1.419745000	2.759142000
H	2.714531000	-3.278639000	1.900781000
H	3.965215000	-2.197200000	1.223108000
H	3.933374000	-4.335206000	0.008511000
H	3.412536000	-3.017753000	-1.076604000
H	1.212102000	-3.698919000	-0.491276000
H	-2.119429000	0.697076000	1.875976000
H	-2.508309000	0.707623000	0.148361000
H	-0.844514000	2.642429000	-0.131195000
H	0.612733000	3.974680000	0.718685000
H	1.128279000	3.749305000	2.417929000
H	0.340751000	5.997255000	2.207646000
H	-0.827845000	5.085877000	3.196603000
H	-2.012628000	4.806557000	1.089405000
H	0.966765000	0.077142000	-0.779964000
H	-0.489223000	0.933040000	-1.289305000
H	-0.277994000	-2.118535000	-1.154024000
H	2.081098000	-1.165922000	-2.096977000
H	1.399195000	-2.738715000	-2.600701000
H	1.464080000	-1.941346000	-4.996348000
H	3.057664000	-2.119957000	-4.202941000
H	2.781752000	-0.080537000	-5.361132000
H	-2.232939000	-1.925727000	-2.497301000

H	-2.253625000	-0.281452000	-2.302595000
H	-3.262643000	2.866757000	1.502323000
H	-3.071426000	3.143844000	-0.109285000
H	0.511281000	-3.405729000	2.052532000
H	-0.508185000	-2.500080000	1.118633000

1E

N	-0.429086000	1.029012000	-0.132531000
C	-0.449802000	2.120087000	0.851934000
C	-1.591983000	2.003094000	1.854599000
O	-1.195245000	1.326499000	2.982513000
C	-2.255523000	0.844127000	3.854376000
C	-2.495537000	-0.638859000	3.672733000
O	-3.080329000	-0.973941000	2.402862000
C	0.591773000	1.318410000	-1.139339000
C	0.594205000	0.319265000	-2.298867000
O	-0.644650000	0.220296000	-2.889371000
C	-0.833864000	-0.944811000	-3.744924000
C	-1.037394000	-2.246985000	-2.994582000
O	0.189106000	-2.809549000	-2.488708000
C	-0.422807000	-0.328442000	0.385273000
C	0.940326000	-0.857170000	0.887350000
O	1.406752000	-0.114154000	1.939911000
C	2.833209000	-0.220918000	2.184947000
C	3.291381000	1.018078000	2.916820000
O	3.170655000	2.216219000	2.132168000
N	0.669882000	-2.331775000	1.319312000
O	0.415133000	-3.141019000	0.413748000
O	0.655647000	-2.605131000	2.517318000
N	1.648371000	0.904700000	-3.321824000
O	1.241597000	1.380991000	-4.378157000
O	2.831009000	0.842888000	-2.978913000
N	-1.997291000	3.494660000	2.251258000
O	-2.535977000	4.169719000	1.373173000

O	-1.768513000	3.874814000	3.395901000
H	-0.583586000	3.047415000	0.280525000
H	0.490526000	2.211072000	1.423471000
H	-2.525142000	1.629501000	1.408556000
H	-3.180368000	1.414941000	3.678362000
H	-1.916901000	1.039459000	4.881190000
H	-3.136010000	-0.984287000	4.503747000
H	-1.542733000	-1.184868000	3.723193000
H	-3.918870000	-0.481039000	2.330192000
H	1.614536000	1.339239000	-0.716999000
H	0.380745000	2.319441000	-1.543446000
H	1.037193000	-0.653819000	-2.050813000
H	0.008519000	-1.029143000	-4.448931000
H	-1.742291000	-0.714968000	-4.316743000
H	-1.468837000	-2.964344000	-3.712894000
H	-1.765780000	-2.107585000	-2.181084000
H	0.190721000	-2.750701000	-1.509763000
H	-0.763291000	-1.007730000	-0.405377000
H	-1.148762000	-0.402717000	1.202456000
H	1.680634000	-0.972629000	0.081438000
H	3.357030000	-0.316716000	1.219222000
H	3.035599000	-1.116835000	2.793355000
H	4.335681000	0.858369000	3.236419000
H	2.679077000	1.177541000	3.815162000
H	3.706165000	2.090985000	1.326164000

1F

N	-0.173501000	-1.602095000	1.102721000
C	-0.315652000	-0.646948000	2.207138000
C	-1.577366000	0.197316000	2.122407000
O	-1.263506000	1.431102000	1.405405000
C	-2.303038000	2.222687000	0.964711000
C	-3.446796000	1.548001000	0.186062000
O	-2.957818000	1.074716000	-1.061542000

C	0.035193000	-0.962243000	-0.202017000
C	-0.599530000	-1.728904000	-1.340241000
O	-2.047844000	-1.652158000	-1.179783000
C	-2.766796000	-2.527380000	-1.957089000
C	-4.167174000	-2.763550000	-1.384145000
O	-4.862062000	-1.574194000	-1.032079000
C	0.785992000	-2.679140000	1.370643000
C	2.251264000	-2.308053000	1.567423000
O	2.457350000	-1.771565000	2.909713000
C	3.162298000	-0.600480000	2.989594000
C	2.905193000	0.066083000	4.348881000
O	3.386008000	1.402442000	4.424459000
N	4.710956000	-0.883556000	2.885664000
O	5.451034000	0.108530000	2.922900000
O	5.099910000	-2.043309000	2.798192000
N	-2.915511000	-1.973638000	-3.422218000
O	-2.648561000	-0.793551000	-3.636680000
O	-3.327749000	-2.772357000	-4.266111000
N	-2.931417000	3.040699000	2.155533000
O	-3.837624000	3.827973000	1.870840000
O	-2.487642000	2.864184000	3.288595000
H	0.548205000	0.039066000	2.312832000
H	-0.368792000	-1.234234000	3.134569000
H	-1.924063000	0.465166000	3.129443000
H	-2.373834000	-0.354443000	1.605776000
H	-1.863887000	3.016199000	0.344218000
H	-4.205893000	2.313912000	-0.014838000
H	-3.912238000	0.746845000	0.777398000
H	-2.662707000	0.139212000	-0.966561000
H	1.106245000	-0.820262000	-0.445228000
H	-0.403594000	0.042158000	-0.181107000
H	-0.310490000	-2.790874000	-1.341301000
H	-0.318582000	-1.278896000	-2.304382000
H	-2.261169000	-3.492463000	-2.114878000

H	-4.021733000	-3.333474000	-0.456690000
H	-4.735612000	-3.384516000	-2.093115000
H	-5.182288000	-1.142895000	-1.845829000
H	0.745962000	-3.384278000	0.526004000
H	0.447296000	-3.236489000	2.258196000
H	2.592540000	-1.569528000	0.827120000
H	2.877205000	-3.206817000	1.472522000
H	2.984647000	0.096434000	2.155107000
H	3.316772000	-0.568121000	5.152389000
H	1.813919000	0.107778000	4.461316000
H	4.346335000	1.376089000	4.237352000

1G

N	-0.173501000	-1.602095000	1.102721000
C	-0.315652000	-0.646948000	2.207138000
C	-1.577366000	0.197316000	2.122407000
O	-1.263506000	1.431102000	1.405405000
C	-2.303038000	2.222687000	0.964711000
C	-3.446796000	1.548001000	0.186062000
O	-2.957818000	1.074716000	-1.061542000
C	0.035193000	-0.962243000	-0.202017000
C	-0.599530000	-1.728904000	-1.340241000
O	-2.047844000	-1.652158000	-1.179783000
C	-2.766796000	-2.527380000	-1.957089000
C	-4.167174000	-2.763550000	-1.384145000
O	-4.862062000	-1.574194000	-1.032079000
C	0.785992000	-2.679140000	1.370643000
C	2.251264000	-2.308053000	1.567423000
O	2.457350000	-1.771565000	2.909713000
C	3.162298000	-0.600480000	2.989594000
C	2.905193000	0.066083000	4.348881000
O	3.386008000	1.402442000	4.424459000
N	4.710956000	-0.883556000	2.885664000
O	5.451034000	0.108530000	2.922900000

O	5.099910000	-2.043309000	2.798192000
N	-2.915511000	-1.973638000	-3.422218000
O	-2.648561000	-0.793551000	-3.636680000
O	-3.327749000	-2.772357000	-4.266111000
N	-2.931417000	3.040699000	2.155533000
O	-3.837624000	3.827973000	1.870840000
O	-2.487642000	2.864184000	3.288595000
H	0.548205000	0.039066000	2.312832000
H	-0.368792000	-1.234234000	3.134569000
H	-1.924063000	0.465166000	3.129443000
H	-2.373834000	-0.354443000	1.605776000
H	-1.863887000	3.016199000	0.344218000
H	-4.205893000	2.313912000	-0.014838000
H	-3.912238000	0.746845000	0.777398000
H	-2.662707000	0.139212000	-0.966561000
H	1.106245000	-0.820262000	-0.445228000
H	-0.403594000	0.042158000	-0.181107000
H	-0.310490000	-2.790874000	-1.341301000
H	-0.318582000	-1.278896000	-2.304382000
H	-2.261169000	-3.492463000	-2.114878000
H	-4.021733000	-3.333474000	-0.456690000
H	-4.735612000	-3.384516000	-2.093115000
H	-5.182288000	-1.142895000	-1.845829000
H	0.745962000	-3.384278000	0.526004000
H	0.447296000	-3.236489000	2.258196000
H	2.592540000	-1.569528000	0.827120000
H	2.877205000	-3.206817000	1.472522000
H	2.984647000	0.096434000	2.155107000
H	3.316772000	-0.568121000	5.152389000
H	1.813919000	0.107778000	4.461316000
H	4.346335000	1.376089000	4.237352000

1H

N	2.072961000	1.624360000	1.021809000

C	2.886341000	0.669279000	1.796672000
C	2.250121000	-0.623195000	2.296266000
O	1.919837000	-1.510223000	1.212467000
C	1.228127000	-2.705996000	1.611065000
C	-0.284008000	-2.577678000	1.395266000
O	-0.479856000	-1.988471000	0.072227000
C	0.671239000	1.648449000	1.445670000
C	-0.146429000	2.772120000	0.816881000
O	-1.417100000	2.871025000	1.490309000
C	-2.571833000	2.434205000	0.765733000
C	-2.751132000	0.917018000	0.700932000
O	-1.889154000	0.407790000	-0.338407000
C	2.285745000	1.606540000	-0.428537000
C	1.654659000	0.455792000	-1.209693000
O	1.839365000	0.726777000	-2.611777000
C	0.934606000	0.037260000	-3.477538000
C	1.396895000	-1.375715000	-3.843468000
O	0.527470000	-1.768817000	-4.925040000
N	1.342611000	-2.369420000	-2.784868000
N	-0.962479000	-1.865356000	2.454333000
N	-4.155731000	0.597084000	0.494749000
H	3.238444000	1.168381000	2.718123000
H	3.783807000	0.432561000	1.207324000
H	1.356466000	-0.400329000	2.899313000
H	2.979347000	-1.122446000	2.959144000
H	1.453591000	-2.964701000	2.656248000
H	1.603386000	-3.508338000	0.960503000
H	-0.717981000	-3.584270000	1.332587000
H	0.277753000	-1.367881000	-0.024932000
H	0.657276000	1.803132000	2.535964000
H	0.144625000	0.694324000	1.252640000
H	-0.306826000	2.614690000	-0.259523000
H	0.366490000	3.738168000	0.953111000
H	-2.561498000	2.843672000	-0.261258000

H	-3.424818000	2.866616000	1.305051000
H	-2.441128000	0.481905000	1.663303000
H	-1.560696000	-0.486969000	-0.071776000
H	3.371783000	1.611925000	-0.604352000
H	1.897069000	2.548202000	-0.847388000
H	2.119939000	-0.500441000	-0.930892000
H	0.572794000	0.404441000	-0.997734000
H	0.874846000	0.638799000	-4.396635000
H	-0.071796000	-0.013153000	-3.021922000
H	2.440850000	-1.328526000	-4.198139000
H	0.699583000	-2.718459000	-5.071430000
H	0.563226000	-2.172041000	-2.148408000
H	2.198185000	-2.326429000	-2.227199000
H	-1.961373000	-1.819693000	2.232559000
H	-0.636327000	-0.893794000	2.484706000
H	-4.246586000	-0.420165000	0.421956000
H	-4.446554000	0.974137000	-0.413796000

1I

N	-0.230004000	-0.344532000	-0.277281000
C	1.210741000	-0.605075000	0.052124000
C	2.040138000	-1.013139000	-1.168876000
O	3.138097000	-1.833275000	-0.715562000
C	3.146743000	-3.176163000	-1.199834000
C	1.888764000	-3.967252000	-0.786335000
O	0.890777000	-3.934907000	-1.722596000
C	-1.002129000	-0.090422000	0.985076000
C	-2.503738000	0.017815000	0.739628000
O	-3.027600000	-0.932480000	-0.195530000
C	-3.249177000	-2.261428000	0.262288000
C	-2.044505000	-3.225122000	-0.005093000
O	-1.130205000	-2.760348000	-0.849263000
C	-0.461555000	0.666946000	-1.363282000
C	-0.143118000	2.102410000	-1.017697000

O	1.283777000	2.293533000	-0.994329000
C	1.675630000	3.637817000	-0.763586000
C	2.318929000	3.858038000	0.614799000
O	2.737217000	5.170512000	0.796940000
N	1.276781000	3.471103000	1.702632000
O	0.483840000	4.336763000	2.074758000
O	1.274052000	2.298228000	2.083398000
N	2.336148000	-5.461593000	-0.537614000
O	1.839423000	-6.350583000	-1.226302000
O	3.176902000	-5.648358000	0.347961000
N	-1.405682000	-3.590420000	1.445538000
O	-2.151306000	-4.146264000	2.261362000
O	-0.227201000	-3.308621000	1.673490000
H	1.201946000	-1.436144000	0.766713000
H	1.619794000	0.280854000	0.549483000
H	2.468438000	-0.135021000	-1.670942000
H	1.422747000	-1.560493000	-1.894460000
H	4.047905000	-3.628173000	-0.769196000
H	3.211751000	-3.200152000	-2.301163000
H	1.557452000	-3.667344000	0.220314000
H	0.007754000	-3.554405000	-1.292215000
H	-0.637342000	0.828573000	1.461111000
H	-0.760865000	-0.927617000	1.648968000
H	-2.752156000	0.997841000	0.311374000
H	-3.013973000	-0.069376000	1.713203000
H	-4.087598000	-2.639510000	-0.337656000
H	-3.547484000	-2.274863000	1.321947000
H	-2.444713000	-4.227674000	-0.248564000
H	-0.629985000	-1.304369000	-0.625967000
H	-1.520769000	0.596908000	-1.635247000
H	0.118992000	0.344134000	-2.236153000
H	-0.594543000	2.399446000	-0.057937000
H	-0.584171000	2.735946000	-1.805207000
H	0.828032000	4.334969000	-0.875859000

H	2.451902000	3.907957000	-1.495361000
H	3.157976000	3.179062000	0.799125000
H	2.055172000	5.775568000	0.441038000

1A···Fe^{2+}

N	-0.094534000	-0.115631000	0.059523000
C	0.868760000	-0.159946000	1.153418000
C	1.268593000	-1.572424000	1.582387000
O	2.253143000	-1.449933000	2.620828000
C	2.917827000	-2.683278000	2.918915000
C	2.029495000	-3.677068000	3.667787000
O	2.774795000	-4.851580000	4.036534000
C	-1.423488000	-0.621003000	0.372293000
C	-2.562461000	0.187889000	-0.248805000
O	-2.447441000	1.647869000	-0.089859000
C	-2.073749000	2.112743000	1.252808000
C	-1.364987000	3.424881000	1.062511000
O	-0.341339000	3.160544000	0.042443000
C	0.461459000	-0.462198000	-1.245549000
C	0.805217000	0.756670000	-2.075568000
O	-0.478578000	1.337485000	-2.495857000
C	-0.484509000	2.484211000	-3.366077000
C	-1.810945000	2.441082000	-4.086288000
O	-2.777241000	2.402647000	-2.977237000
H	0.449984000	0.373188000	2.021716000
H	1.769767000	0.392721000	0.844537000
H	1.697639000	-2.134894000	0.733119000
H	0.384904000	-2.123882000	1.946037000
H	3.777757000	-2.417713000	3.553131000
H	3.298134000	-3.143966000	1.988529000
H	1.207122000	-4.030969000	3.031504000
H	1.594778000	-3.188412000	4.557021000
H	3.461803000	-4.572394000	4.670193000
H	-1.534821000	-0.638561000	1.464668000

H	-1.592697000	-1.664013000	0.035668000
H	-3.521291000	-0.109761000	0.199566000
H	-2.615067000	0.045194000	-1.333905000
H	-1.411492000	1.383569000	1.726427000
H	-2.991182000	2.231857000	1.843509000
H	-0.881253000	3.748522000	1.992970000
H	-2.037975000	4.219601000	0.701565000
H	0.145089000	3.991040000	-0.138861000
H	-0.253752000	-1.079456000	-1.810250000
H	1.382736000	-1.062535000	-1.149712000
H	1.329132000	0.484442000	-3.002190000
H	1.376082000	1.511872000	-1.519047000
H	-0.410505000	3.440406000	-2.706142000
H	-1.914651000	1.553018000	-4.718392000
H	-1.984342000	3.357379000	-4.661784000
H	-3.252972000	1.546819000	-2.989605000
Fe	-1.490667000	2.604509000	-1.467816000
H	0.411732000	2.557487000	-3.995137000

1A···Zn^{2+}

N	0.144600000	0.103547000	1.205418000
C	0.770477000	-0.948648000	2.019196000
C	0.196194000	-2.361645000	1.832660000
O	1.047684000	-3.351113000	2.436769000
C	2.128834000	-3.737538000	1.581525000
C	3.045139000	-4.644700000	2.381545000
O	4.108403000	-5.056921000	1.503708000
C	-1.278972000	0.253544000	1.521585000
C	-1.921949000	1.495602000	0.937646000
O	-1.343848000	2.689482000	1.425216000
C	-1.647394000	3.838037000	0.659643000
C	-3.100681000	4.237223000	0.520843000
O	-3.749119000	3.442617000	-0.554333000
C	0.400373000	-0.055217000	-0.238906000

C	1.036849000	1.204342000	-0.826698000
O	1.061005000	1.110990000	-2.263705000
C	0.518348000	2.218855000	-2.979739000
C	-0.631683000	1.813564000	-3.860597000
O	-1.794788000	1.391365000	-3.027826000
H	0.685181000	-0.664805000	3.079158000
H	1.842047000	-0.962292000	1.773797000
H	0.056464000	-2.593388000	0.762085000
H	-0.777749000	-2.459272000	2.331385000
H	2.692091000	-2.855950000	1.228104000
H	1.735480000	-4.270567000	0.696097000
H	2.480274000	-5.518416000	2.750111000
H	3.444817000	-4.096292000	3.251292000
H	4.724177000	-5.595751000	2.031295000
H	-1.390538000	0.299903000	2.615554000
H	-1.895239000	-0.594517000	1.161574000
H	-3.010851000	1.465893000	1.091625000
H	-1.724465000	1.439054000	-0.186870000
H	-1.207188000	3.711121000	-0.373995000
H	-1.091053000	4.669338000	1.111267000
H	-3.161273000	5.289361000	0.224003000
H	-3.673302000	4.053812000	1.437427000
H	-4.434639000	3.975808000	-1.009661000
H	-0.527868000	-0.279117000	-0.794498000
H	1.072165000	-0.908689000	-0.408530000
H	2.069053000	1.320241000	-0.456341000
H	0.476008000	2.097878000	-0.514870000
H	1.287394000	2.644859000	-3.647691000
H	0.219558000	3.029698000	-2.289658000
H	-0.389985000	0.932766000	-4.466709000
H	-0.949036000	2.647948000	-4.497955000
H	-2.451643000	0.935866000	-3.594629000
Zn	-2.562704000	2.423224000	-1.639642000

1$_B$···Zn^{2+}

Zn	-0.727315000	0.157820000	0.646027000
O	-1.124923000	-1.794906000	0.334663000
O	-1.672490000	0.387168000	-1.244715000
O	-2.295785000	0.527899000	1.958904000
O	0.397860000	0.373734000	2.454529000
O	0.912713000	-3.383561000	0.273143000
O	2.345259000	-1.311426000	-1.531719000
N	2.950162000	1.717571000	-0.946939000
N	0.210924000	3.341171000	1.624125000
N	0.356466000	3.392214000	-1.860575000
N	0.616437000	1.559830000	-0.120580000
C	-1.966177000	-1.938655000	-0.844542000
C	-1.591783000	-0.916372000	-1.891323000
C	-1.066245000	1.469749000	-1.986435000
C	-0.269935000	2.383033000	-1.046929000
C	-1.824074000	0.097479000	3.275672000
C	-0.472794000	0.701522000	3.576544000
C	1.475442000	1.315134000	2.193054000
C	1.110771000	2.339170000	1.117192000
C	1.555216000	-3.604371000	-1.016547000
C	2.722720000	-2.662592000	-1.218256000
C	2.091093000	-0.502236000	-0.383359000
C	1.749572000	0.897666000	-0.897239000
H	3.762514000	1.115951000	-1.092805000
H	2.913680000	2.379919000	-1.723080000
H	0.115633000	4.124693000	0.977193000
H	-0.726409000	2.985079000	1.825790000
H	0.896126000	4.049767000	-1.294666000
H	0.974463000	2.976798000	-2.561707000
H	-0.306986000	-2.411192000	0.257223000
H	-3.002493000	-1.797598000	-0.510970000
H	-1.850733000	-2.951579000	-1.249954000
H	-2.292679000	-0.950758000	-2.737641000

H	-0.561849000	-1.071715000	-2.249858000
H	-1.862590000	2.062525000	-2.454020000
H	-0.427306000	1.055361000	-2.780455000
H	-0.986446000	2.870280000	-0.374995000
H	-2.538835000	1.474832000	2.006322000
H	-1.769472000	-0.996246000	3.214336000
H	-2.555235000	0.387051000	4.039988000
H	-0.052485000	0.266031000	4.494431000
H	-0.541592000	1.791580000	3.688246000
H	2.332371000	0.708627000	1.880090000
H	1.739065000	1.833394000	3.122156000
H	2.044698000	2.832350000	0.822464000
H	0.506911000	-4.226669000	0.548863000
H	0.815417000	-3.476283000	-1.822495000
H	1.935633000	-4.635668000	-1.045896000
H	3.288265000	-3.021495000	-2.090377000
H	3.391321000	-2.677222000	-0.340305000
H	2.979022000	-0.493067000	0.273442000
H	1.261740000	-0.926025000	0.205696000
H	1.319285000	0.730213000	-1.894933000

1c····Zn^{2+}

N	-0.138269000	-0.604073000	-1.240802000
C	-1.116177000	0.373114000	-1.760216000
C	-0.964820000	0.830988000	-3.202285000
O	0.331806000	1.404222000	-3.255887000
C	0.512081000	2.204963000	-4.439345000
C	1.922316000	2.729288000	-4.469987000
O	2.831339000	1.638420000	-4.707178000
C	0.003278000	-1.812320000	-2.053772000
C	1.421389000	-2.243363000	-2.498422000
O	1.909802000	-1.389206000	-3.508460000
C	1.835651000	-1.920190000	-4.853552000
C	0.536820000	-1.636884000	-5.576803000

O	-0.619180000	-2.263411000	-4.987983000
C	1.078021000	0.024773000	-0.677045000
C	0.748477000	1.235416000	0.209945000
O	-0.396129000	0.901091000	1.010932000
C	-0.311122000	1.177296000	2.441341000
C	-1.524975000	0.535328000	3.057230000
O	-1.502702000	-0.893180000	2.701747000
N	1.768946000	-0.950568000	0.309198000
O	2.978392000	-0.930566000	0.365593000
O	1.037636000	-1.633268000	1.060771000
N	-0.662440000	-3.050781000	-1.361050000
O	-1.119117000	-2.990539000	-0.192397000
O	-0.715727000	-4.059978000	-2.027242000
N	-2.553295000	-0.141687000	-1.494469000
O	-3.407964000	0.061972000	-2.323760000
O	-2.778721000	-0.684314000	-0.386560000
H	-1.098248000	1.262636000	-1.122777000
H	-1.740754000	1.587256000	-3.399182000
H	-1.087744000	0.011146000	-3.928468000
H	-0.195870000	3.051584000	-4.421521000
H	0.304597000	1.594821000	-5.335964000
H	2.156793000	3.229831000	-3.515064000
H	1.989415000	3.477530000	-5.278485000
H	3.734544000	2.000818000	-4.670302000
H	-0.598444000	-1.745291000	-2.975538000
H	1.344880000	-3.281366000	-2.848310000
H	2.130227000	-2.230804000	-1.661735000
H	2.033083000	-3.005044000	-4.821218000
H	2.652954000	-1.434875000	-5.406095000
H	0.661170000	-1.964421000	-6.624487000
H	0.328342000	-0.559051000	-5.582287000
H	-0.433850000	-3.218881000	-4.907931000
H	1.846174000	0.266964000	-1.417895000
H	0.533279000	2.112098000	-0.415121000

H	1.622020000	1.466424000	0.833471000
H	-0.333990000	2.262237000	2.606317000
H	0.624310000	0.750540000	2.830951000
H	-2.453426000	0.979815000	2.676411000
H	-1.479985000	0.620460000	4.149481000
H	-2.327847000	-1.314080000	3.014204000
Zn	-1.063726000	-1.150621000	0.772672000

1$_{D}$···Zn^{2+}

N	-0.015241000	-0.349563000	0.632007000
C	0.430404000	-0.263124000	2.033925000
C	0.725994000	-1.637526000	2.636386000
O	1.921640000	-2.111702000	1.950835000
C	2.428886000	-3.393414000	2.338969000
C	2.785431000	-4.171954000	1.085686000
O	1.636101000	-4.518614000	0.300390000
C	-1.079865000	0.593774000	0.298439000
C	-0.640917000	2.074742000	0.225429000
O	-1.335166000	2.827174000	1.193032000
C	-0.660670000	4.021925000	1.660649000
C	-0.839910000	5.242499000	0.785705000
O	-0.013963000	5.134011000	-0.399923000
C	1.096209000	-0.369162000	-0.317082000
C	0.904635000	-1.251656000	-1.541516000
O	0.133959000	-0.431627000	-2.607955000
C	0.406840000	-0.866226000	-3.973370000
C	-0.391091000	0.012144000	-4.899928000
O	-0.080653000	1.407770000	-4.530639000
N	0.258504000	-2.465625000	-1.254164000
N	-0.901506000	2.630718000	-1.151933000
N	-0.427617000	-2.492428000	2.525723000
H	-0.376339000	0.185918000	2.626185000
H	1.319063000	0.382886000	2.147353000
H	0.967883000	-1.515539000	3.705414000

H	1.686093000	-3.958327000	2.924492000
H	3.329152000	-3.262515000	2.966549000
H	3.272758000	-5.114769000	1.376635000
H	3.505663000	-3.590605000	0.482726000
H	1.265613000	-3.690617000	-0.103672000
H	-1.870076000	0.510482000	1.054822000
H	-1.541334000	0.281946000	-0.652245000
H	0.443754000	2.172398000	0.387532000
H	0.409890000	3.798976000	1.803335000
H	-1.108369000	4.234508000	2.640321000
H	-0.532740000	6.125794000	1.369248000
H	-1.899015000	5.357313000	0.505069000
H	-0.238732000	5.880834000	-0.985139000
H	1.968375000	-0.783793000	0.198645000
H	1.413414000	0.642069000	-0.652380000
H	1.871471000	-1.423354000	-2.030108000
H	1.487167000	-0.779306000	-4.158885000
H	0.085526000	-1.909624000	-4.094731000
H	-1.471407000	-0.146457000	-4.789617000
H	-0.081815000	-0.147345000	-5.939274000
H	-0.648147000	2.017836000	-5.044109000
H	0.130027000	-3.064359000	-2.070653000
H	-0.615621000	-2.319534000	-0.744851000
H	-1.922475000	2.675032000	-1.290480000
H	-0.543758000	3.617974000	-1.162961000
H	-0.250853000	-3.404340000	2.949892000
H	-0.633949000	-2.640774000	1.534180000
Zn	-0.200023000	1.511125000	-2.562376000

1E···Zn^{2+}

N	0.124025000	1.050430000	-0.206662000
C	0.034361000	2.129000000	0.710578000
C	-1.212049000	2.080637000	1.599624000
O	-1.074823000	1.183638000	2.632036000

C	-2.301990000	0.622682000	3.176293000
C	-2.039009000	-0.811511000	3.582103000
O	-1.771910000	-1.674470000	2.468786000
C	0.628846000	1.385962000	-1.531478000
C	0.633370000	0.222041000	-2.510321000
O	-0.643701000	-0.155637000	-2.822255000
C	-0.795502000	-1.506840000	-3.353096000
C	-2.193471000	-1.974902000	-3.025878000
O	-2.407959000	-2.175871000	-1.619376000
C	0.047321000	-0.298401000	0.246365000
C	1.403379000	-0.782594000	0.856793000
O	1.520190000	-0.437544000	2.165804000
C	2.781294000	0.027441000	2.641825000
C	2.620230000	1.387865000	3.308210000
O	2.378507000	2.460140000	2.435996000
N	1.355944000	-2.369264000	0.742499000
O	1.327387000	-2.814464000	-0.399419000
O	1.374682000	-3.013379000	1.779374000
N	1.393584000	0.765912000	-3.792897000
O	0.773200000	0.816467000	-4.847484000
O	2.572616000	1.080594000	-3.634004000
N	-1.367960000	3.515590000	2.251840000
O	-2.511529000	3.800224000	2.603245000
O	-0.364230000	4.194448000	2.443146000
H	0.069149000	3.074041000	0.158631000
H	0.940962000	2.125180000	1.404217000
H	-2.140852000	1.950832000	1.026864000
H	-3.095928000	0.683451000	2.416417000
H	-2.595155000	1.214160000	4.057050000
H	-2.916680000	-1.162013000	4.151259000
H	-1.158398000	-0.868391000	4.235893000
H	-2.567722000	-1.687318000	1.903475000
H	1.658726000	1.773055000	-1.427056000
H	0.011497000	2.207532000	-1.927415000

H	1.286135000	-0.608737000	-2.196979000
H	-0.039457000	-2.159106000	-2.889289000
H	-0.643249000	-1.483440000	-4.442544000
H	-2.350611000	-2.948178000	-3.511226000
H	-2.932353000	-1.264904000	-3.433755000
H	-2.397540000	-1.299635000	-1.191564000
H	-0.200266000	-0.957664000	-0.589877000
H	-0.704439000	-0.411979000	1.042870000
H	2.262229000	-0.505380000	0.229944000
H	3.526026000	0.058547000	1.833177000
H	3.116510000	-0.689577000	3.423509000
H	3.606612000	1.568142000	3.846838000
H	1.830871000	1.386129000	4.069174000
H	3.051657000	2.452821000	1.720422000
Zn	5.054419000	0.237258000	4.803319000

1$_F$···Zn^{2+}

N	0.721727000	0.219510000	0.199277000
C	0.435677000	-1.236537000	0.023381000
C	-0.641579000	-1.703233000	0.985803000
O	-1.830678000	-0.871431000	0.902559000
C	-2.924890000	-1.304752000	-0.030935000
C	-2.678491000	-0.665745000	-1.388023000
O	-2.475539000	0.762641000	-1.167607000
C	1.582041000	0.475964000	1.405831000
C	0.935001000	1.274488000	2.525876000
O	0.841130000	2.694960000	2.241976000
C	-0.409409000	3.303852000	1.977111000
C	-1.601811000	2.839112000	2.815816000
O	-2.210798000	1.661810000	2.216098000
C	1.318511000	0.820831000	-1.038236000
C	2.619609000	0.214638000	-1.537191000
O	2.349484000	-0.977560000	-2.280842000
C	3.545556000	-1.621550000	-2.757811000

C	3.091579000	-2.638396000	-3.794524000
O	2.357760000	-3.684262000	-3.129816000
N	4.307177000	-2.190801000	-1.655511000
N	-0.762653000	3.101872000	0.542389000
N	-4.186563000	-0.945459000	0.508990000
H	1.344982000	-1.834756000	0.181004000
H	0.111352000	-1.388900000	-1.012301000
H	-0.307937000	-1.627599000	2.029712000
H	-0.909254000	-2.747654000	0.775694000
H	-2.833064000	-2.395116000	-0.113103000
H	-3.551921000	-0.803978000	-2.036954000
H	-1.790530000	-1.093174000	-1.868673000
H	-2.224871000	1.182266000	-2.013133000
H	2.495736000	0.994840000	1.088413000
H	1.891838000	-0.488529000	1.832205000
H	1.589473000	1.192255000	3.405427000
H	-0.035393000	0.847865000	2.811258000
H	-0.240542000	4.370510000	2.178381000
H	-2.354691000	3.638546000	2.823725000
H	-1.287307000	2.626746000	3.843838000
H	-2.293579000	0.953235000	2.881128000
H	1.500158000	1.880110000	-0.818282000
H	0.562778000	0.765920000	-1.835993000
H	3.310923000	-0.000519000	-0.706499000
H	3.111344000	0.953176000	-2.197382000
H	4.175110000	-0.868742000	-3.263260000
H	3.984835000	-3.046942000	-4.292939000
H	2.461146000	-2.141924000	-4.551868000
H	2.338337000	-4.450974000	-3.728538000
H	4.027114000	-3.167250000	-1.526369000
H	5.301669000	-2.183561000	-1.888520000
H	0.014000000	3.425925000	-0.041560000
H	-1.586938000	3.666432000	0.302863000
H	-4.295784000	0.070365000	0.564176000

H	-4.306582000	-1.339989000	1.443501000
Zn	-1.182034000	1.099316000	0.402781000

1$_{G}\cdots Zn^{2+}$

N	0.392896000	-0.815863000	0.691611000
C	0.339646000	0.488711000	1.428487000
C	-0.955321000	0.770708000	2.165050000
O	-1.856200000	1.540279000	1.328189000
C	-3.222549000	1.366325000	1.489529000
C	-3.775153000	-0.029081000	1.209838000
O	-3.759244000	-0.168210000	-0.210815000
C	-0.432895000	-0.765552000	-0.565649000
C	-0.976542000	-2.121270000	-0.975546000
O	-1.729329000	-2.589912000	0.198861000
C	-2.250387000	-3.872741000	0.233484000
C	-2.309059000	-4.372236000	1.714305000
O	-1.767756000	-3.389383000	2.640087000
C	1.813322000	-1.183017000	0.385003000
C	2.590634000	-1.548414000	1.639463000
O	1.809505000	-2.488642000	2.480848000
C	2.297422000	-3.790520000	2.602911000
C	1.905741000	-4.344042000	3.994331000
O	2.466630000	-5.612446000	4.261762000
N	1.648420000	-4.675791000	1.524719000
O	0.578053000	-4.274993000	1.002935000
O	2.153171000	-5.751085000	1.282225000
N	-3.680882000	-3.901380000	-0.362347000
O	-4.153791000	-2.883157000	-0.863612000
O	-4.239097000	-4.992462000	-0.309261000
N	-3.705568000	1.863657000	2.896224000
O	-4.901879000	1.727976000	3.155901000
O	-2.878911000	2.386846000	3.640312000
H	0.568545000	1.303734000	0.724950000
H	1.136415000	0.465839000	2.178912000

H	-0.727417000	1.370761000	3.054584000
H	-1.440177000	-0.157854000	2.512174000
H	-3.709169000	2.082938000	0.812348000
H	-4.799390000	-0.095562000	1.605308000
H	-3.163075000	-0.804885000	1.695877000
H	-3.716284000	-1.128850000	-0.409833000
H	0.167583000	-0.346174000	-1.385373000
H	-1.278527000	-0.096052000	-0.375097000
H	-0.201846000	-2.865921000	-1.206655000
H	-1.660175000	-2.010867000	-1.824867000
H	-1.704603000	-4.576585000	-0.408903000
H	-1.680607000	-5.260383000	1.814549000
H	-3.333721000	-4.604686000	2.016524000
H	-2.478307000	-2.766017000	2.901526000
H	1.792862000	-2.034113000	-0.307171000
H	2.787995000	-0.692112000	2.291066000
H	3.539948000	-2.020351000	1.363345000
H	3.374843000	-3.865334000	2.419631000
H	0.809615000	-4.338837000	4.096415000
H	2.335127000	-3.620421000	4.700832000
H	1.845747000	-6.302821000	3.963492000
Zn	-0.230861000	-2.420893000	1.766229000
H	2.320962000	-0.341796000	-0.111898000

1$_{H}\cdots Zn^{2+}$

N	1.051578000	0.701190000	-0.027547000
C	2.339196000	0.933206000	0.696209000
C	2.679737000	-0.235859000	1.591625000
O	2.761466000	-1.419489000	0.738459000
C	2.602497000	-2.697060000	1.397864000
C	1.141683000	-3.068710000	1.716014000
O	0.312520000	-2.583127000	0.574983000
C	-0.146632000	0.754297000	0.863572000
C	-0.330950000	2.050132000	1.658589000

O	-1.595685000	1.988770000	2.339352000
C	-2.631629000	2.836897000	1.823480000
C	-3.313082000	2.354976000	0.542819000
O	-2.401702000	2.580112000	-0.539812000
C	0.905685000	1.629415000	-1.191865000
C	0.161386000	0.959954000	-2.328071000
O	0.948725000	-0.210227000	-2.635979000
C	0.410677000	-1.138590000	-3.588224000
C	1.350336000	-2.337659000	-3.539923000
O	0.811181000	-3.327102000	-4.393565000
N	1.463485000	-2.803757000	-2.133125000
N	0.593327000	-2.580378000	2.942717000
N	-4.569841000	3.048683000	0.274509000
H	2.313419000	1.856730000	1.292077000
H	3.122417000	1.046977000	-0.065256000
H	1.919361000	-0.383240000	2.368236000
H	3.656873000	-0.082638000	2.068135000
H	3.205233000	-2.717577000	2.315591000
H	3.010502000	-3.423264000	0.684585000
H	1.063850000	-4.162462000	1.674964000
H	-0.499834000	-2.226428000	0.988464000
H	-0.092951000	-0.081694000	1.571925000
H	-1.027433000	0.592727000	0.230277000
H	-0.281964000	2.941734000	1.017813000
H	0.441607000	2.143995000	2.435724000
H	-2.247504000	3.861734000	1.663492000
H	-3.391475000	2.868390000	2.616519000
H	-3.535371000	1.279037000	0.636019000
H	-2.785744000	2.161874000	-1.333272000
H	1.912819000	1.891233000	-1.539347000
H	0.396781000	2.557393000	-0.901889000
H	-0.860776000	0.655309000	-2.051833000
H	0.119481000	1.625345000	-3.201990000
H	0.410429000	-0.694708000	-4.592982000

H	-0.614920000	-1.430932000	-3.305522000
H	2.357883000	-2.034822000	-3.858353000
H	1.529750000	-3.935395000	-4.648660000
H	0.671194000	-3.425292000	-1.926861000
H	2.320486000	-3.350650000	-2.003552000
H	0.261807000	-3.337441000	3.537442000
H	1.230706000	-1.986930000	3.470009000
H	-5.280394000	2.709475000	0.929849000
H	-4.447426000	4.048900000	0.470475000
Zn	1.294500000	-1.270473000	-0.761609000

$1_{I}\cdots Zn^{2+}$

N	-0.593004000	0.189905000	-1.233543000
C	0.459532000	-0.564697000	-0.737118000
C	1.717916000	-0.745956000	-1.571288000
O	2.643056000	-1.629068000	-0.928511000
C	2.172968000	-2.950790000	-0.649594000
C	1.664537000	-3.666674000	-1.902078000
O	2.699113000	-3.873186000	-2.793927000
C	-1.837193000	0.074300000	-0.472613000
C	-2.521484000	-1.290062000	-0.581565000
O	-1.882416000	-2.344348000	0.172291000
C	-2.408682000	-2.516702000	1.480046000
C	-1.340396000	-3.069727000	2.418156000
O	-0.678194000	-4.196925000	2.027996000
C	-0.347593000	1.453590000	-1.931951000
C	-0.061113000	2.661111000	-1.051324000
O	1.277953000	2.580405000	-0.527766000
C	1.559604000	3.698106000	0.295563000
C	2.873862000	3.460648000	1.046418000
O	3.106565000	4.336427000	2.075417000
N	2.711197000	1.981161000	1.577828000
O	1.837350000	1.778807000	2.473195000
O	3.242625000	1.039220000	1.004325000

N	1.061217000	-5.033742000	-1.418052000
O	1.584209000	-6.073423000	-1.798840000
O	0.067063000	-4.971752000	-0.684116000
N	-0.280296000	-1.895754000	2.615260000
O	-0.654611000	-0.733799000	2.816134000
O	0.930482000	-2.116809000	2.354423000
H	0.073040000	-1.518473000	-0.364879000
H	0.861432000	-0.032187000	0.282438000
H	2.263051000	0.194492000	-1.704329000
H	1.430622000	-1.125820000	-2.565852000
H	1.381564000	-2.957454000	0.111884000
H	3.044118000	-3.491008000	-0.256659000
H	0.786449000	-3.178559000	-2.348519000
H	2.328536000	-4.034025000	-3.683266000
H	-2.536028000	0.829570000	-0.853327000
H	-1.641209000	0.304507000	0.595998000
H	-2.529116000	-1.629269000	-1.625223000
H	-3.564112000	-1.180072000	-0.248843000
H	-3.210472000	-3.275864000	1.480809000
H	-2.809358000	-1.582336000	1.896647000
H	-1.751523000	-3.194169000	3.429228000
H	-0.458623000	-4.150914000	1.067260000
H	-1.251752000	1.679002000	-2.513591000
H	0.474226000	1.312224000	-2.646089000
H	-0.777727000	2.730035000	-0.214932000
H	-0.160250000	3.570585000	-1.669094000
H	0.758933000	3.850431000	1.043206000
H	1.687555000	4.624282000	-0.292352000
H	3.757217000	3.384340000	0.404057000
H	2.263998000	4.561558000	2.523123000
Zn	1.354350000	-0.127658000	1.975672000
